# Targeting adaptor protein SLP76 of RAGE as a therapeutic approach for lethal sepsis

**DOI:** 10.1038/s41467-020-20577-3

**Published:** 2021-01-12

**Authors:** Zhengzheng Yan, Haihua Luo, Bingyao Xie, Tian Tian, Shan Li, Zhixia Chen, Jinghua Liu, Xuwen Zhao, Liyong Zhang, Yongqiang Deng, Timothy R. Billiar, Yong Jiang

**Affiliations:** 1 Guangdong Provincial Key Laboratory of Proteomics, State Key Laboratory of Organ Failure Research, Department of Pathophysiology, School of Basic Medical Sciences, Southern Medical University, Guangzhou, 510515 China; 2 Department of Surgery, University of Pittsburgh School of Medicine, Pittsburgh, PA 15213 USA; 3 Department of Anesthesiology, Nanfang Hospital, Southern Medical University, Guangzhou, 510515 China

**Keywords:** Cell signalling, Monocytes and macrophages, Bacterial infection

## Abstract

Accumulating evidence shows that RAGE has an important function in the pathogenesis of sepsis. However, the mechanisms by which RAGE transduces signals to downstream kinase cascades during septic shock are not clear. Here, we identify SLP76 as a binding partner for the cytosolic tail of RAGE both in vitro and in vivo and demonstrate that SLP76 binds RAGE through its sterile α motif (SAM) to mediate downstream signaling. Genetic deficiency of RAGE or SLP76 reduces AGE-induced phosphorylation of p38 MAPK, ERK1/2 and IKKα/β, as well as cytokine release. Delivery of the SAM domain into macrophages via the TAT cell-penetrating peptide blocks proinflammatory cytokine production. Furthermore, administration of TAT-SAM attenuates inflammatory cytokine release and tissue damage in mice subjected to cecal ligation and puncture (CLP) and protects these mice from the lethality of sepsis. These findings reveal an important function for SLP76 in RAGE-mediated pro-inflammatory signaling and shed light on the development of SLP76-targeted therapeutics for sepsis.

## Abstract (web summary)

RAGE signalling is implicated in sepsis. Here the authors use T7 phage display to identify SLP76 as a binding partner for the cytosolic tail of RAGE and provide a reagent that can block this interaction and protect mice from sepsis in a caecal ligation and puncture model.

## Introduction

Sepsis is a main cause of death in noncoronary intensive care units (ICUs) and is characterized as life-threatening infection with organ dysfunction^1,2^. Despite extensive studies on its pathogenesis and therapeutic strategies and improvements in its clinical treatment, the incidence and mortality of sepsis remain high^3–5^. Sepsis is characterized by an overwhelming activation of innate immunity by a large family of pattern recognition receptors (PRRs), including Toll-like receptors (TLRs), the receptor for advanced glycation end products (RAGE)^6^, etc., in response to infectious pathogens. During sepsis development, proinflammatory mediators, including cytokines, chemokines, growth factors, and damage-associated molecular pattern (DAMP) molecules generated by a variety of cells, trigger strong immune responses that result in organ dysfunction or failure and even death. TLRs have been shown to play a pivotal role in the initiation of systemic inflammatory responses to invaded pathogens; however, various therapeutic strategies targeting TLRs or downstream signaling molecules to block systemic inflammation have failed in clinical trials^7,8^.

Advanced glycation end products (AGEs) are generated through nonenzymatic glycation and oxidation of proteins, lipids, and nucleic acids^9^. AGEs are reported to be involved in the progression of many diseases, including Alzheimer’s disease (AD), diabetes mellitus (DM), chronic inflammatory diseases, and even some acute diseases^10–12^. Recently, AGEs were reported to accumulate at high concentrations in the heart and lung tissues of patients with sepsis, leading to tissue injury by binding with the multiligand transmembrane receptor RAGE^13,14^.

As a type I immunoglobulin superfamily member, RAGE consists of an extracellular region, a transmembrane region, and a short 43-amino acid cytosolic tail (CT)^15–17^. Plasma soluble RAGE (sRAGE) has been observed at high concentrations in septic patients, indicating that RAGE plays an important role in the pathophysiological processes of sepsis^18,19^. Blocking RAGE activation by various techniques, such as *AGER* (encoding RAGE protein) gene knockout (KO), small interfering RNA (siRNA) targeting RAGE mRNA, sRAGE, and specific neutralizing antibodies, confers beneficial effects on organ damage as well as prolongs the survival of mice subjected to cecal ligation and puncture (CLP)^20–22^.

The biological function of a receptor is usually mediated by conformational changes in its cytoplasmic domain. Previous in vitro and in vivo studies have demonstrated that the RAGE cytoplasmic domain is critical for RAGE ligand-stimulated cell responses^23^. The cytoplasmic domain was also found to be necessary for RAGE-mediated activation of intracellular signaling cascades. Although the RAGE cytoplasmic domain lacks endogenous tyrosine kinase activity, cytoplasmic domain-deficient RAGE was found to lose the capability to activate cells. This finding strongly suggesting that RAGE interacts with one or more cytoplasmic binding proteins to trigger the recruitment of downstream signaling molecules, thus mediating downstream signaling transduction. The formin homology (FH1) domain of mammalian diaphanous 1 (DIAPH1, also named mDia1) was identified as a binding partner for the short cytoplasmic tail of RAGE (ctRAGE), through which RAGE mediates Rho GTPase (Cdc42 and Rac-1) activation and cell migration^15,20^. Although the RAGE/mDia/GTPase/cytoskeleton axis explains the induction of cell migration by RAGE ligands in some cells, the intermediate signaling events responsible for the RAGE binding-induced proinflammatory response are unclear.

In the present study, we use the T7 phage display system to screen for proteins interacting with the cytosolic tail of RAGE and identified Src homology 2 domain-containing leukocyte protein of 76 kDa (SLP76) as an adaptor protein for the transduction of RAGE-mediated signals to downstream effectors in macrophages. Furthermore, we prove that the sterile α motif (SAM) domain of SLP76 is a functional domain for the interaction with RAGE. Importantly, blocking the interaction between RAGE and SLP76 with the TAT-SAM fusion peptide not only inhibits the downstream signaling events, but also decreases the cytokine release induced by RAGE activation, thus prolonging the survival time of mice subjected to CLP.

## Results

### Screening of the RAGE-binding peptides by T7 phage display biopanning

**Fig. 1:**
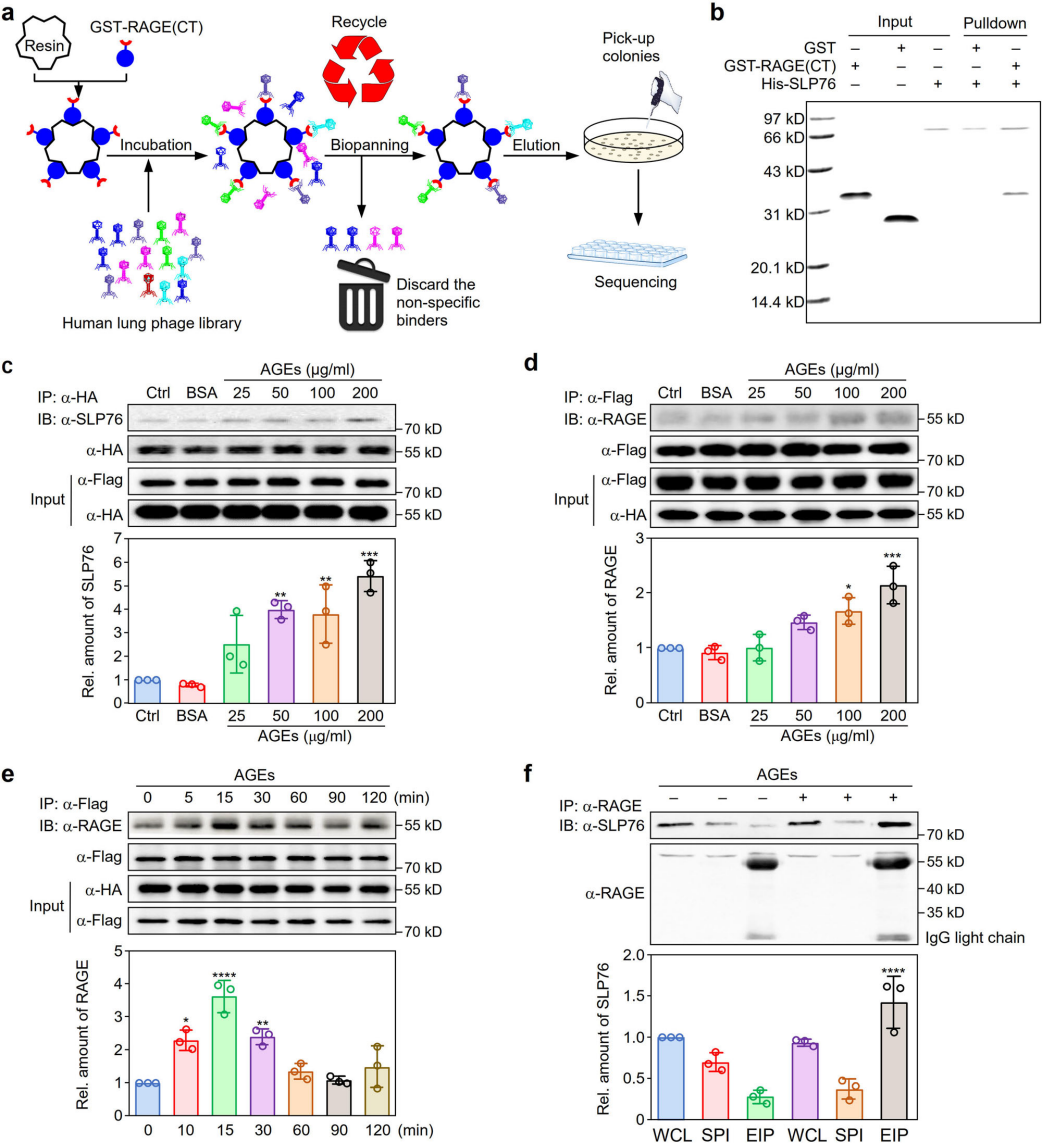
Identification of SLP76 as a binding protein of the RAGE intracellular domain. **a** Schematic diagram showing the T7 phage display system strategy used to screen for the potential proteins binding with the cytosolic tail of RAGE. **b** In vitro binding of the cytoplasmic domain of RAGE with SLP76. In vitro binding was assessed with recombinant GST-RAGE(CT) and His-SLP76 proteins. GST was used as the control. The experiment was repeated three times with similar results. **c** Dose-dependent interaction between RAGE and SLP76. Plasmids expressing Flag-tagged SLP76 and HA-tagged RAGE were cotransfected into HEK293 cells. Twenty-four hours after transfection, cells were treated with different doses of AGEs. Co-IP was performed with a specific HA-tag antibody (*n* = 3). **P < 0.01, ***P < 0.001 versus the control group. **d** Reverse binding of RAGE to SLP76. The interaction between SLP76 and RAGE was further confirmed by the procedure described in (**c**) with a specific antibody against the Flag tag for reverse immunoprecipitation (*n* = 3). *P < 0.05, ***P < 0.001 versus the control group. **e** Time-dependent interaction between RAGE and SLP76. After cotransfection with plasmids expressing Flag-SLP76 and HA-RAGE, HEK293 cells were treated with AGEs (100 μg/ml) for different times to assess the interaction between SLP76 and RAGE (*n* = 3). *P < 0.05, **P < 0.01, ****P < 0.0001 versus the control group. **f** Endogenous interaction between RAGE and SLP76. After stimulation with AGEs (100 μg/ml) for 15 min, RAW264.7 cells were lysed for immunoprecipitation with a specific antibody against RAGE to confirm the endogenous interaction between RAGE and SLP76. The efficiency of Co-IP for RAGE and SLP76 was determined by the intensity ratio of protein bands of SLP76 and RAGE, which was normalized by the corresponding protein bands of input. For SDS-PAGE, the loading volume of the whole-cell lysate (WCL) and supernatant post immunoprecipitation (SPI) was 20 μl (1/20 of input) (*n* = 3). EIP: eluates from the immunoprecipitated protein G beads. ****P < 0.0001 versus the untreated EIP group. Data are presented as mean ± SD in (**c**–**f**). Statistics were performed using one-way ANOVA (**c**–**f**) followed by Tukey post hoc. Source data are provided as a Source data file.

RAGE is constitutively expressed on the surface of most immune cells, and many experimental studies with gene knockout mice or specific inhibitors have demonstrated that blockade of RAGE reduces the production of various proinflammatory mediators implicated in sepsis pathology and prolongs the survival time of mice subjected to CLP^24^. To elucidate the mechanism of the RAGE-mediated signaling process and its importance in the pathogenesis of sepsis, we performed biopanning with the T7 phage display system to identify the potential proteins interacting with the cytosolic tail of RAGE (Fig. 1a). After three rounds of biopanning, T7 phage clones were collected from the eluate. The pfu values of recovered bacteriophage in rounds 1–3 were 2.0 × 10^9^, 1.2 × 10^8^, and 1.4 × 10^8^ pfu, respectively, although the amount of input bacteriophage was the same (2.0 × 10^11^ pfu) in every round (Supplementary Fig. 1a). Correspondingly, the bacteriophage enrichment ratios remained relatively stable after 2 rounds of biopanning (Supplementary Fig. 1b). By DNA sequencing of enriched bacteriophages, we identified 11 candidate proteins as binding partners for the cytosolic tail of RAGE (Supplementary Table 1). Based on the functional and structural analysis results, we focused our further study on the adaptor protein SLP76.

### Interaction between SLP76 and the cytoplasmic domain of RAGE

The cytoplasmic domain of RAGE is reported to be crucial for activation of intracellular signaling cascades by RAGE ligands^20^. To confirm the binding activity of SLP76 to RAGE, recombinant glutathione S-transferase (GST), GST-tagged cytosolic tail of RAGE (GST-RAGE(CT)), and His-tagged SLP76 (His-SLP76) proteins were purified from bacteria (Supplementary Fig. 2). The fusion protein His-SLP76 was incubated with GST or GST-RAGE(CT), and a pulldown assay was performed with Ni^2+^-NTA resin to assess the interaction between SLP76 and RAGE. The coimmobilization assay results demonstrated that GST-RAGE(CT), but not GST, was pulled down by His-SLP76 (Fig. 1b), indicating that SLP76 interacts with the cytosolic tail of RAGE. To further clarify the interaction between RAGE and SLP76 in vivo, after treatment with different doses of AGEs, coimmunoprecipitation (Co-IP) experiments were performed with HA-coupled (Fig. 1c) or Flag-coupled (Fig. 1d) beads in HEK293 cells cotransfected with plasmids expressing HA-tagged RAGE and Flag-tagged SLP76. The AGE-induced interaction between RAGE and SLP76 was also observed at different times (Fig. 1e). These results indicated that the binding of RAGE and SLP76 after exposure to AGEs is dose- and time-dependent (Fig. 1c–e). Co-IP was performed to evaluate the interaction between endogenous SLP76 and RAGE in RAW264.7 cells. SLP76 was predominantly precipitated by a specific antibody against RAGE after stimulation with AGEs for 15 min (Fig. 1f). These results confirmed that SLP76 is a binding partner for the cytosolic domain of RAGE.

### Identification of the binding domains of RAGE and SLP76

**Fig. 2:**
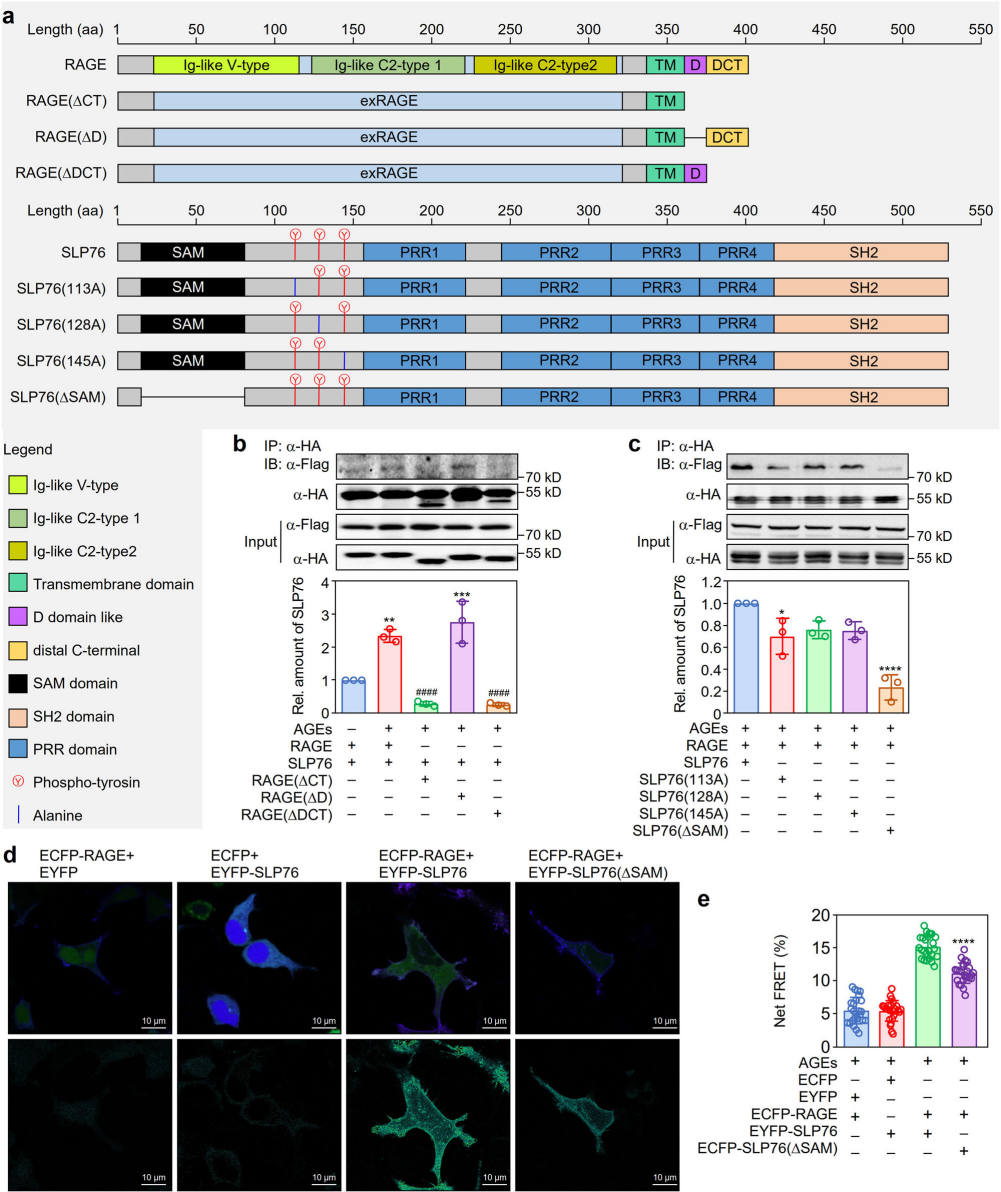
Identification of the functional domains that mediate the interaction between RAGE and SLP76. **a** Schematic diagram of the protein domains of RAGE and SLP76. The specific domains/motifs of RAGE (upper panel) and SLP76 (lower panel) are shown in distinct colors. Different truncations or mutants of RAGE and SLP76 were constructed and are displayed in the upper and lower panels, respectively. **b** Mapping the region of RAGE that binds with SLP76. Flag-tagged full-length SLP76 and HA-tagged WT RAGE or its deletion mutants were overexpressed in HEK293 cells. After treatment with or without AGEs (100 μg/ml) for 15 min, cells were collected for lysis. Immunoprecipitation was performed with a specific antibody against the HA tag to detect the interaction between RAGE and SLP76 (*n* = 3). **P < 0.01, ***P < 0.001 versus untreated cells, ^####^*P* < 0.0001 versus AGE-treated cells cotransfected with full-length RAGE and SLP76. **c** Identification of the functional domain of SLP76 that interacts with RAGE. HA-tagged full-length RAGE and Flag-tagged WT SLP76 or its mutants were overexpressed in HEK293 cells. Immunoprecipitation with a specific antibody against the HA tag was performed to identify the domain of SLP76 interacting with RAGE (*n* = 3). *P < 0.05, ****P < 0.0001 versus cells coexpressing full-length RAGE and SLP76. **d** The SAM domain mediates the in vivo interaction between RAGE and SLP76. Plasmids expressing ECFP-tagged RAGE and EYFP-tagged SLP76 or SLP76(ΔSAM) were coexpressed in HEK293 cells. FRET assays were performed to verify the binding activity of the SAM domain of SLP76 with RAGE (*n* = 5). **e** Quantification of the in vivo interaction of RAGE and SLP76 (*n* = 5). The FRET efficiency was further quantitated by the formula Net FRET (N-FRET) = FRET signal − (a*YFP signal) − (b*CFP signal) as described in the “Methods”. The results for each group are shown. ****P < 0.0001 versus cells cotransfected with full-length RAGE and SLP76. Data are presented as mean ± SD in (**b**), (**c**), and (**e**). Statistics were performed using one-way ANOVA (**b**, **c**, and **e**) followed by Tukey post hoc. Scale bar: 10 μm. Source data are provided as a Source data file.

Both RAGE and SLP76 have many functional domains (Fig. 2a). To identify the interaction domains in RAGE and SLP76, several RAGE mutants, including mutants with cytosolic tail deletion, D-domain-like docking site (D) deletion, and distal C terminal (DCT) domain deficiency (the cytosolic part of RAGE without the D-domain-like docking site), were generated. The Co-IP assay results showed that the cytosolic tail domain of RAGE was essential for the binding of RAGE with SLP76. Furthermore, the DCT region of RAGE was indispensable for binding with the adaptor protein SLP76 (Fig. 2b). Parallel experiments with phosphotyrosine site mutants (Y^113^A, Y^128^A, and Y^145^A) and a SAM domain deletion mutant (ΔSAM) of SLP76 demonstrated that SAM domain deletion markedly decreased the interaction between SLP76 and RAGE (Fig. 2c). Consistent with this result, the fluorescence resonance energy transfer (FRET) assay results also showed that SAM domain deficiency markedly decreased the efficiency of fluorescence energy transfer (Fig. 2d–e). To determine the binding affinity of the SAM domain of SLP76 and the cytosolic tail of RAGE, surface plasmon resonance imaging (SPRi) assay was conducted with the synthetic peptides. We found that there was a dose-dependent binding between the SAM domain of SLP76 and the cytosolic tail of RAGE (Supplementary Fig. 3). Collectively, these findings indicated that the interaction between SLP76 and RAGE was mediated by the SAM domain of SLP76 and the DCT region of RAGE.

### Effect of AGEs on the proinflammatory signaling pathways in macrophages

**Fig. 3:**
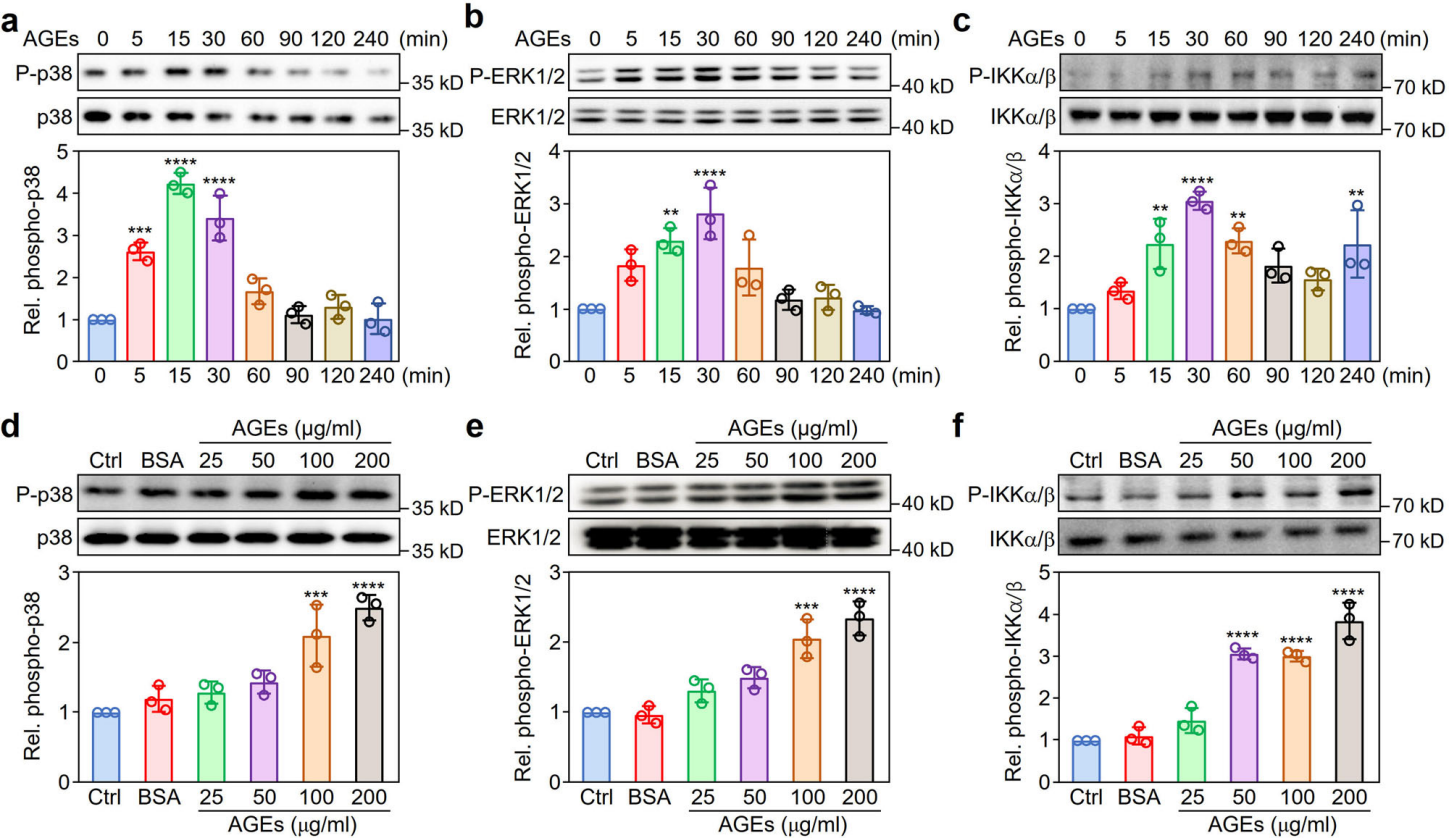
Effect of AGEs on proinflammatory signaling pathways in macrophages. **a**–**c** Activation dynamics of p38 MAPK, ERK1/2, and IKKα/β in RAW264.7 cells induced by AGEs. After treatment with AGEs (100 μg/ml) for different times (0–240 min), RAW264.7 cells were harvested for western blotting with specific antibodies against phosphorylated (upper panel) or total (lower panel) p38 MAPK (**a**), ERK1/2 (**b**), or IKKα/β (**c**). **d**–**f** Dose-dependent activation of p38 MAPK, ERK1/2, and IKKα/β in RAW264.7 cells stimulated with AGEs. RAW264.7 cells were treated with AGEs at the indicated doses for 15 min. Western blotting was performed with specific antibodies to detect phosphorylation of p38 MAPK (**d**), ERK1/2 (**e**), and IKKα/β (**f**). Protein band densities were quantified with ImageJ software and are presented as fold changes (FC) in the phosphorylated kinase relative to the corresponding total kinases. Data from three independent experiments (*n* = 3) are shown as the mean ± SD values. **P < 0.01, ***P < 0.001, ****P < 0.0001 versus untreated cells (one-way ANOVA plus Tukey post hoc). Source data are provided as a Source data file.

To explore the mechanistic details of downstream signaling events mediated by RAGE in the treatment of AGEs, we performed western blotting with specific antibodies against phosphorylated kinases and found that the mitogen-activated protein kinase (MAPK) and IKKα/β signaling pathways were dynamically activated in RAW264.7 cells (Fig. 3). However, RAGE-mediated activation of MAPK pathways might be selective; indeed, our results demonstrated that phosphorylation of p38 MAPK and ERK1/2 (Fig. 3a–c) but not JNKs (Supplementary Fig. 4) increased significantly after treatment with AGEs for 15 min (*P* < 0.05). To investigate the effects of dose-dependent activation of RAGE by AGEs, we incubated RAW264.7 cells with different doses of AGEs for 15 min and found that phosphorylation of p38 MAPK, ERK1/2, and IKKα/β was dramatically enhanced as the concentration of AGEs increased (Fig. 3d–f).

Collectively, these results indicated that activation of the proinflammatory signaling pathways, including the p38 MAPK, ERK1/2, and IKKα/β pathways, induced by AGE stimulation was a dose- and time-dependent process in macrophages.

### Indispensable role of SLP76 in RAGE-mediated signaling

**Fig. 4:**
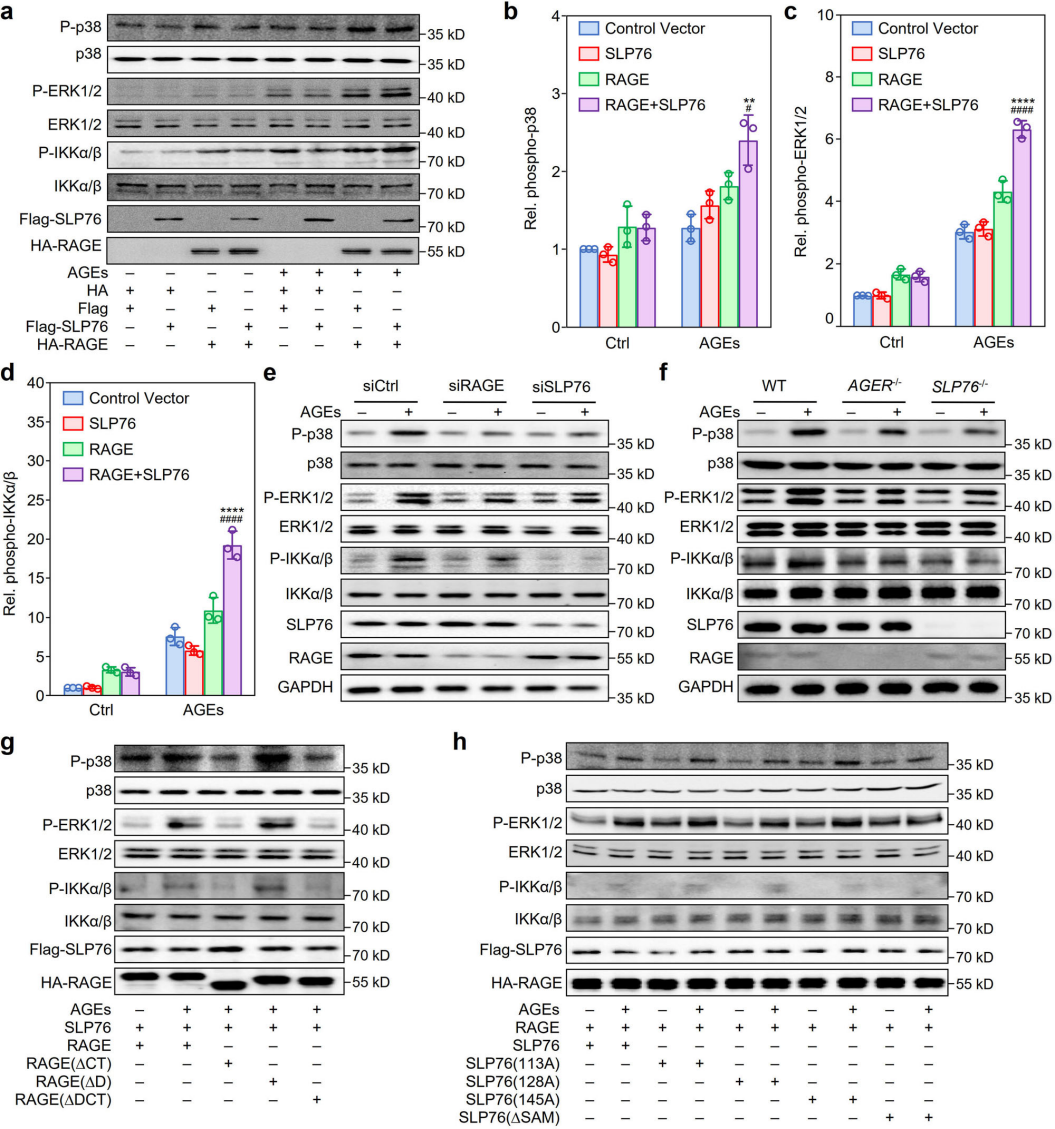
Function of SLP76 in RAGE-mediated activation of proinflammatory signaling pathways. **a** Effects of RAGE and SLP76 overexpression on proinflammatory signaling pathways in HEK293 cells treated with AGEs. Flag-tagged SLP76 and HA-tagged RAGE were overexpressed together or separately in HEK293 cells treated with or without AGEs (100 μg/ml) for 15 min. Plasmids expressing a Flag or HA tag were used as controls. Phosphorylation of MAPKs and IKKα/β was detected by western blotting (*n* = 3). **b**–**d** Quantification of phosphorylated protein kinases activated by treatment with AGEs. The levels of phosphorylated p38 MAPK (**b**), ERK1/2 (**c**), and IKKα/β (**d**) were quantified with ImageJ software. Data were from three independent experiments (*n* = 3) and are shown as the mean ± SD. **P < 0.01, ****P < 0.0001 versus the group transfected with SLP76 alone, ^####^*P* < 0.0001 versus cells transfected with RAGE alone (one-way ANOVA plus Tukey post hoc). **e** Effects of RAGE and SLP76 on AGE-induced activation of p38, ERK1/2, and IKKα/β in RAW264.7 cells. After transfection with control siRNA or with specific siRNA targeting *AGER* or *SLP76* for 48 h, RAW264.7 cells were stimulated with AGEs (100 μg/ml) for 15 min (*n* = 3). **f** BMDMs isolated from WT, *AGER* knockout (*AGER*^*−/−*^), and *SLP76* knockout (*SLP76*^*−/−*^) mice were treated with AGEs (100 μg/ml) for 15 min. Cells were harvested for detection of p38 MAPK, ERK1/2, and IKKα/β phosphorylation. The results represent three independent experiments (*n* = 3). **g** Effect of the intracellular region of RAGE on AGE-induced activation of signaling pathways. Flag-tagged full-length SLP76 and HA-tagged WT RAGE or RAGE deletion mutants were overexpressed in HEK293 cells. After treatment with AGEs (100 μg/ml) for 15 min, cells were harvested to detect phosphorylation of p38 MAPK, ERK1/2, and IKKα/β (*n* = 3). **h** Role of the SLP76 domain interacting with RAGE in AGE-induced activation of p38 MAPK, ERK1/2, and IKKα/β. HEK293 cells were cotransfected with plasmids expressing HA-tagged RAGE and Flag-tagged WT SLP76 or mutant SLP76. After transfection for 24 h, cells were treated as indicated. Western blotting was performed to detect phosphorylation of p38 MAPK, ERK1/2, and IKKα/β (*n* = 3). Source data are provided as a Source data file.

To assess the role of SLP76 in RAGE-mediated cell activation, we transfected plasmids expressing HA-tagged RAGE or Flag-tagged SLP76 into HEK293 cells and performed western blotting with specific antibodies to detect phosphorylation of signaling molecules after stimulation with AGEs for 15 min. Cells with overexpression of RAGE or SLP76 alone exhibited only slight or even no changes in AGE-induced phosphorylation of p38 MAPK, ERK1/2, and IKKα/β, but cells with coexpression of RAGE and SLP76 exhibited dramatically enhanced phosphorylation of these signaling molecules (Fig. 4a–d).

To prove that the interaction between RAGE and SLP76 is responsible for AGE-induced RAW264.7 cell activation, we used siRNA to silence endogenous SLP76 or RAGE mRNA expression in RAW264.7 cells (Supplementary Fig. 5a, b). Phosphorylation of p38 MAPK, ERK1/2, and IKKα/β was significantly decreased when the gene expression of RAGE and SLP76 was knocked down (Fig. 4e). Parallel experiments with bone marrow-derived macrophages (BMDMs) demonstrated that genetic deficiency of either *AGER* or *SLP76* prominently repressed AGE-induced phosphorylation of p38 MAPK, ERK1/2, and IKKα/β (Fig. 4f).

To further investigate the effects of RAGE and SLP76 mutants on kinase phosphorylation, RAGE or SLP76 mutants were overexpressed in HEK293 cells by transfection. Interestingly, when the DCT of RAGE or SAM domain of SLP76 was deleted, AGE-induced phosphorylation of p38 MAPK, ERK1/2, and IKKα/β was significantly suppressed (Fig. 4g, h).

Bioinformatic analysis revealed that the short cytosolic region of RAGE contains several potential phosphorylation sites, including Y^391^, Y^399^, Y^400^, and T^401^. RAGE-mediated signaling was not significantly affected in cells overexpressing the tyrosine phosphorylation site mutants of RAGE compared with those overexpressing wild-type (WT) RAGE (Supplementary Fig. 6).

In summary, these results demonstrated that SLP76 was critical for RAGE activation-mediated cell signaling and that the SAM domain of SLP76 and the DCT of RAGE were required not only for the interaction between RAGE and SLP76 but also for signal transduction mediated by the AGE/RAGE axis.

### Role of SLP76 in AGE-induced cytokine and chemokine release

To explore the role of SLP76 in RAGE-mediated cytokine and chemokine release, specific siRNAs targeting *AGER* (siRAGE) or *SLP76* (siSLP76) were synthesized. We measured cytokine levels via enzyme-linked immunosorbent assay (ELISA) and found that release of TNF and IL-6 was significantly suppressed by silencing the expression of RAGE or SLP76 compared with that in the control siRNA (siCtrl) group (Supplementary Fig. 7a, b).

**Fig. 5:**
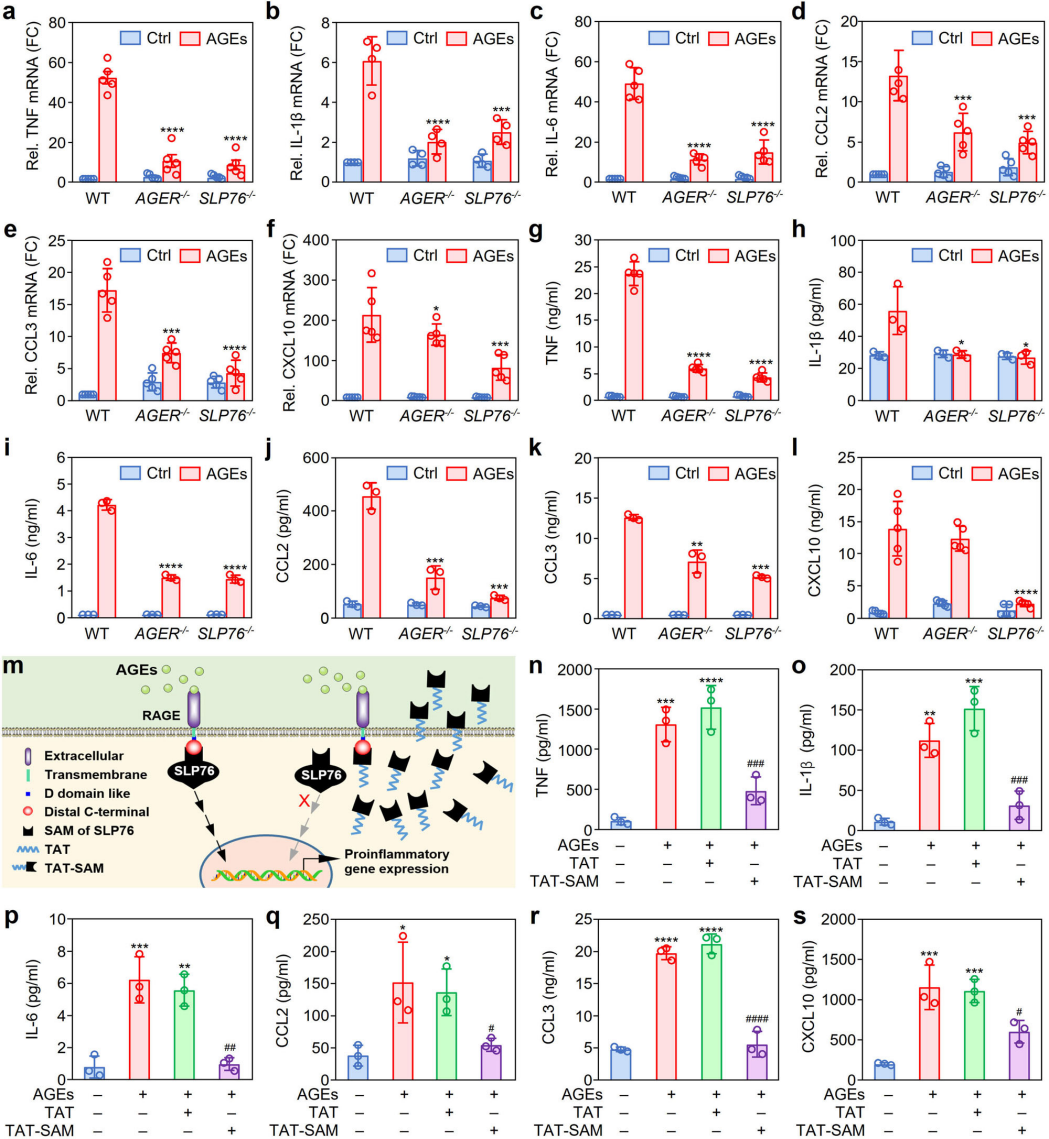
Function of SLP76 in RAGE-mediated cytokine and chemokine expression. **a**–**f** Inhibitory effect of *AGER* and *SLP76* gene deficiency on the mRNA expression of proinflammatory cytokines and chemokines. BMDMs were isolated from WT, *AGER*^*−/−*^, or *SLP76*^*−/−*^ mice and were then stimulated with or without AGEs for 3 h. The levels of mRNAs encoding cytokines and chemokines in BMDMs were quantitated by real-time PCR (*n* = 5). *P < 0.05, ***P < 0.001, ****P < 0.0001 compared with the WT group (two-way ANOVA with Tukey post hoc). **g**–**l** Effect of *AGER* and *SLP76* gene deficiency on cytokine and chemokine production in BMDMs. BMDMs were isolated from WT, *AGER*^*−/−*^, or *SLP76*^*−/−*^ mice and were then stimulated with or without AGEs (100 μg/ml) for 12 h. The cytokines and chemokines in culture media were quantitated by using a multiplex LiquiChip system (*n* = 3). *P < 0.05, **P < 0.01, ***P < 0.001, ****P < 0.0001 compared with the WT group (two-way ANOVA with Tukey post hoc). **m** The blocking mechanism of the TAT-SAM peptide on RAGE-mediated cell signaling. After AGEs bind with the cell surface receptor RAGE, SLP76 is recruited to the cytosolic tail of RAGE as an adaptor protein to transduce signals from the extracellular environment into the cell. A peptide containing the SAM domain was delivered into cells via the TAT CPP, thus blocking the interaction between RAGE and endogenous SLP76 through competitive binding with the cytosolic tail of RAGE, which resulted in inhibition of the signaling process mediated by RAGE. **n**–**s** Effects of the synthetic TAT-SAM peptide on AGE-induced production of cytokines and chemokines. RAW264.7 cells were pretreated with the TAT-SAM peptide or with TAT alone for 1 h and were then subjected to stimulation with AGEs for 12 h. The levels of cytokines and chemokines in culture media were quantitated by using a multiplex LiquiChip system (*n* = 3). *P < 0.05, **P < 0.01, ***P < 0.001, ****P < 0.0001 compared with the untreated group; ^#^*P* < 0.05, ^##^*P* < 0.01, ^###^*P* < 0.001, ^####^*P* < 0.0001 compared with the TAT group (one-way ANOVA with Tukey post hoc). Data are presented as mean ± SD in (**a**–**i**) and (**n**–**s**). Source data are provided as a Source data file.

To confirm the above results, *SLP76* gene knockout mice were designed and generated by Cyagen Biosciences (Guangzhou, China) (Supplementary Fig. 8). BMDMs were isolated from WT mice and *AGER*- or *SLP76*-deficient mice to characterize the expression profile of proinflammatory genes and the cytokine release induced by AGEs. Genetic deficiency of *AGER* and *SLP76* resulted in significant decreases in both the expression levels of proinflammatory genes, including *Tnf*, *Il-1b*, *Il6*, *Ccl2*, *Ccl3*, and *Cxcl10* (Fig. 5a–f), and the release of the corresponding cytokines and chemokines (Fig. 5g–l).

The above results reasonably suggest that disruption of the signaling pathways mediated by SLP76 might result in reduced production of proinflammatory cytokines. Thus, we used specific inhibitors to investigate the roles of signaling pathways in the release of proinflammatory cytokines. Correspondingly, we found that specific inhibitors of p38 MAPK, ERK1/2, and IKKα/β significantly decreased the release of TNF and IL-6 into the supernatant of cultured cells (Supplementary Fig. 9).

Previous studies have demonstrated that the TAT cell-penetrating peptide (CPP) can enter a variety of cell lines with high efficiency^25,26^. Thus, the TAT-SAM fusion peptide was commercially synthesized (Supplementary Fig. 10) to deliver the SAM domain of SLP76 into RAW264.7 cells (Fig. 5m). The delivery efficiency of TAT was evaluated by fluorescence microscopy with a TAT-EGFP fusion peptide. As shown in Supplementary Fig. 11a, b, TAT-mediated delivery of EGFP into RAW264.7 cells was dose-dependent. After pretreatment with the TAT peptide, TAT-SAM, or TAT-RAGE(DCT) for 1 h, RAW264.7 cells were stimulated with AGEs. Phosphorylation of p38 MAPK, ERK1/2, and IKKα/β was prominently inhibited in the TAT-SAM and TAT-RAGE(DCT) groups compared with the TAT group (Supplementary Fig. 11c–e). Consistent with this result, administration of the TAT-SAM peptide dramatically inhibited the release of proinflammatory cytokines and chemokines from AGE-treated RAW264.7 cells (Fig. 5n–s).

Taken together, these findings indicated that blocking the interaction between RAGE and SLP76 with the TAT-SAM peptide not only inhibited the proinflammatory signaling pathways but also suppressed AGE-induced cytokine release.

### SAM domain of SLP76 as a potential therapeutic target for sepsis

**Fig. 6:**
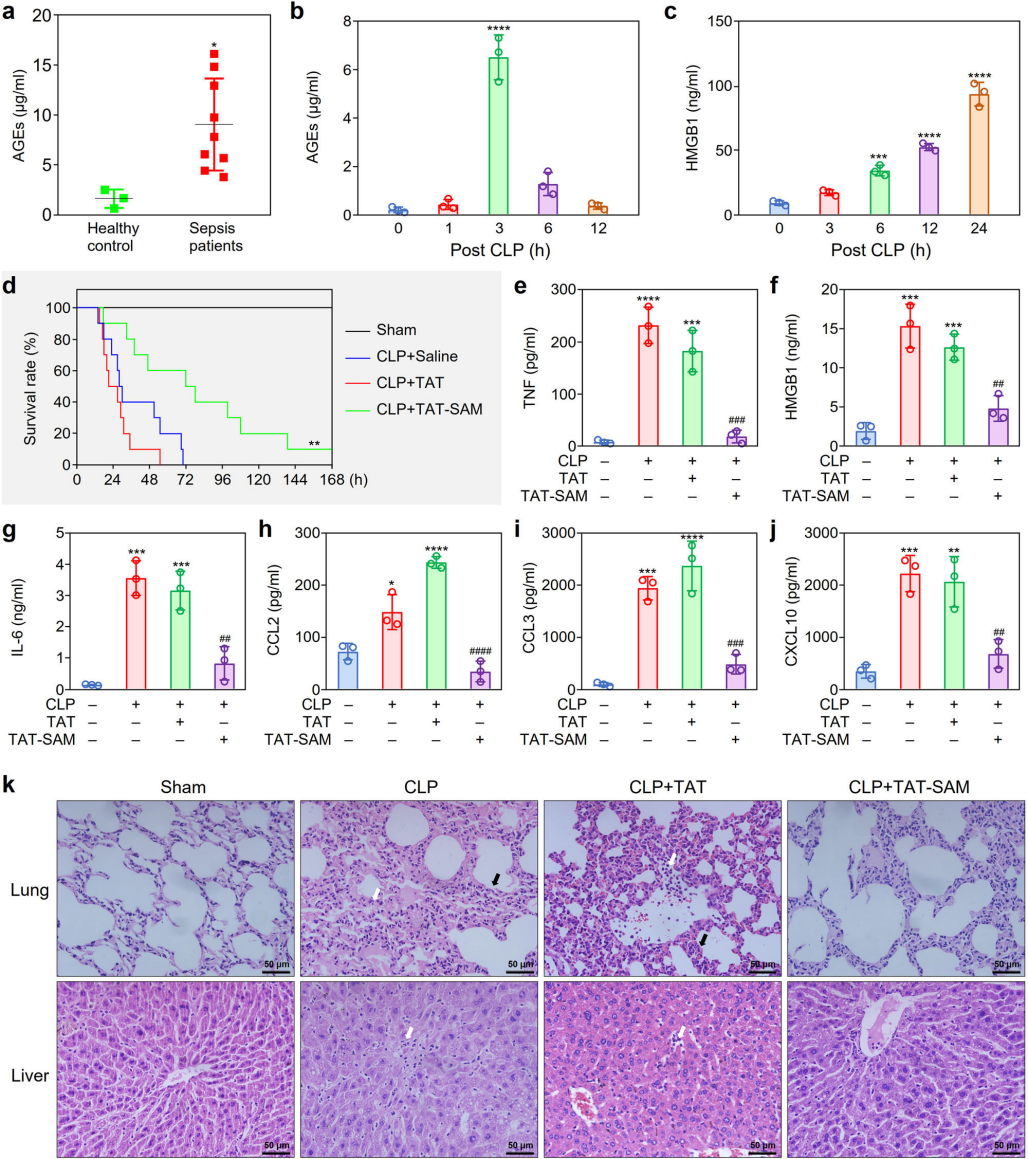
Effects of the synthetic TAT-SAM peptide on survival and cytokine production in septic mice. **a** Elevation of AGEs in the serum of patients with sepsis. The AGEs in the serum of sepsis patients (*n* = 9) and healthy controls (*n* = 3) were measured by ELISA. *P < 0.05 compared with control. **b** Time-dependent increase of AGEs in the serum of septic mice. The AGEs in the serum of CLP mice was quantitated by ELISA (*n* = 3). ****P < 0.0001 compared with the 0 h group. **c** Time-dependent accumulation of HMGB1 in the serum of septic mice. The HMGB1 in the serum of CLP mice was measured by ELISA (*n* = 3). ***P < 0.001, ***^*^P < 0.0001 compared with the 0 h group. **d** The therapeutic effect of the TAT-SAM peptide on the survival of septic mice. One hour after CLP, mice were subjected to TVI with normal saline, TAT (10 μM) or the TAT-SAM (10 μM) peptide. The survival of mice was observed for 7 days. Kaplan–Meier survival analysis was performed to evaluate the survival of septic mice. **P < 0.01 compared with the CLP + Saline group (*n* = 10). **e**–**j** Inhibitory effect of the TAT-SAM peptide on the production of cytokines and chemokines in septic mice. The serum of mice was collected 12 h post CLP. The cytokines and chemokines were measured by using a multiplex LiquiChip system. *P < 0.05, **P < 0.01, ***P < 0.001, ****P < 0.0001 compared with the sham group; ^##^*P* < 0.01, ^###^*P* < 0.001, ^####^*P* < 0.0001 compared with the CLP + TAT group (*n* = 3). **k** Protective effects of the TAT-SAM peptide against lung and liver tissue injury in septic mice. Lung and liver tissues were collected from mice 12 h after CLP. H&E staining of the lung tissue sections showed massive infiltration of inflammatory cells (white arrows), marked interalveolar septal thickening and alveolar edema (black arrows). The liver tissue sections showed extensive liver damages and inflammatory cell infiltration (white arrows) (*n* = 5). Data are presented as mean ± SD in *P*-values by two-tailed unpaired *t* test in (**a**), one-way ANOVA in (**b**), (**c**), and (**e**–**j**) followed by Tukey post hoc. Scale bar: 50 μm. Source data are provided as a Source data file.

Previous studies demonstrated that an increase in AGEs in the blood was associated with various critical illnesses^13^. Our data showed that AGEs accumulated not only in patients with sepsis but also in mice subjected to CLP (Fig. 6a, b). Interestingly, after the levels of AGEs decreased, the level of the DAMP molecule high mobility group box 1 protein (HMGB1) was prominently elevated 6 h after CLP and increased further until the end of the 24-h observation period (Fig. 6c).

As a functional ligand for RAGE, HMGB1 has been reported to be a key factor in the development of sepsis^27,28^. We found that HMGB1 treatment also activated proinflammatory signaling pathways via SLP76 in RAW264.7 cells, which induced long-lasting activation of RAGE-mediated signaling (Supplementary Fig. 12).

Based on the above findings, we aimed to evaluate whether the TAT-SAM peptide protects mice from septic shock in a CLP model. Intriguingly, tail vein injection (TVI) of TAT-SAM 1 h after CLP significantly reduced the mortality rate of CLP model mice. Intriguingly, administration of TAT-SAM through TVI 1 h after CLP modeling reduced the mortality of septic mice in a dose-dependent manner (Supplementary Fig. 13). Administration of TAT-SAM (10 µM), but not the TAT peptide, could significantly improve the survival of septic mice (Fig. 6d). Consistent with this finding, serum analysis showed that TAT-SAM-treated mice had lower serum concentrations of various proinflammatory cytokines than control mice (Fig. 6e–j). Furthermore, histopathological examination showed that administration of the TAT-SAM peptide had a therapeutic effect on inflammatory infiltration and tissue damage in the lung and liver tissues of mice subjected to CLP (Fig. 6k). Similarly, we found that gene deficiency of *SLP76* or *AGER* significantly reduced the levels of cytokines in the blood and tissue damage. Notably, there is no significant difference on cytokine production and pathophysiological examination between TAT-SAM-treated WT mice and *AGER* or *SLP76* gene knockout mice (Supplementary Fig. 14).

## Discussion

Sepsis is a life-threatening disorder associated with prolonged inflammation, immune suppression, and organ injury. Controlling the progression of infections with antibiotics is the main therapeutic strategy for sepsis and septic shock^29^. Recent approaches aimed at inhibiting the bioactivity of bacterial components or proinflammatory mediators have been suggested to treat patients with sepsis; however, none of these methods have consistent satisfactory therapeutic effects in patients with sepsis^30^. Based on these limitations, the development of new therapeutic strategies for sepsis is urgently needed.

AGEs have long been considered potent toxic molecules that promote host cell death and contribute to tissue damage and organ failure. AGEs have been reported to be involved in the development of various diseases, including diabetic complications, cardiovascular diseases (CVDs), and neurodegenerative diseases, as well as in aging^9,10^. In addition, high plasma levels of AGEs were observed in patients with sepsis^13^. Consistent with a previous study, our study showed that the concentration of AGEs in plasma was higher in septic patients than in healthy controls (Fig. 6a).

RAGE is a multiligand cell surface receptor and plays a pivotal role in inflammation and infection. Binding of AGEs with RAGE initiates distinct signaling cascades that contribute to the pathogenesis of infectious diseases^31^. However, the signaling pathways mediated by RAGE are very complex and are characterized by numerous ligands (AGEs, HMGB1, and S100 proteins) and broad receptor expression across many cell types, including endothelial cells, myeloid cells, lymphocytes, and tumor cells. The cellular response mediated by RAGE is dependent not only on the cell type, the identity and concentration of the ligand, the surface amount of RAGE, and the presence of putative coreceptors but also on the different adaptor proteins mediating the intracellular signaling pathways.

The role of RAGE in sepsis has been demonstrated in many studies^32–34^. For example, *AGER*-deficient mice were proven to exhibit protection from lethal polymicrobial sepsis caused by CLP^35^. Moreover, in mice subjected to CLP modeling, either blocking RAGE with a neutralizing antibody or silencing RAGE expression with a specific siRNA decreased proinflammatory cytokine release and resulted in improved survival^34,35^. As a secretory isoform of RAGE, sRAGE contains the extracellular binding domain but lacks the cytosolic and transmembrane domains. Importantly, sRAGE can abrogate RAGE-mediated cell signaling by competitive interaction with its ligands^36,37^. Reports have indicated that the plasma level of sRAGE is significantly increased in patients with sepsis and that the sRAGE level in nonsurvivors is higher than that in survivors^18^. Given this important role of RAGE in diseases, illustration of RAGE-mediated signaling is helpful not only for our understanding of the molecular mechanisms underlying sepsis pathogenesis but also for the development of therapeutic strategies.

The cytosolic portion of RAGE consists of 43 amino acids and does not contain any known functional domains or motifs for cellular signal transduction. Previous studies demonstrated that a RAGE mutant lacking the intracellular tail failed to activate downstream signaling and to induce the release of proinflammatory cytokines from macrophages, indicating a crucial role of the cytoplasmic tail of RAGE in transducing extracellular signals into cells^20,38^.

Because the cytosolic portion is important for RAGE-mediated signaling, several laboratories have tried to characterize this short intracellular region. Ishihara et al. reported that ERK1/2 directly associated with RAGE by binding with the D-domain-like docking site located in the cytosolic domain of RAGE^39^, but they did not further investigate the role of ERK1/2 in RAGE-mediated signaling activation. Hudson et al. demonstrated that mDia1 binding to ctRAGE is essential for RAGE ligand-stimulated phosphorylation of AKT and cell proliferation/migration^20^. Although many studies have demonstrated that the cytosolic tail of RAGE is critical for RAGE ligand-induced cell activation^15,40,41^, the role of the membrane-proximal region in signaling is largely unclear^20^.

In the present study, we identified the adaptor protein SLP76 as a binding partner for the cytosolic tail of RAGE by using the T7 phage display system. As a hematopoietic cell-specific adaptor protein, SLP76 functions as a regulator in multiple signaling pathways downstream of surface receptor binding in platelets^42^, mast cells^43^, and neutrophils^44^. SLP76 is composed of four key structures: an SH2 domain, a proline-rich region, a SAM domain, and an acidic domain containing three tyrosine phosphorylation sites (Y^113^, Y^128^, and Y^145^ in humans)^43,45^. SLP76 was reported to function as a scaffold protein to form microclusters with Grap2, Vav1, and Nck, which were subsequently recruited to the membrane-proximal region by LAT to integrate signals for T-cell activation^46^. Despite the importance of SLP76 in T-cell immunity, the expression and function of SLP76 in macrophages are not well characterized.

To identify the functional region in the cytosolic tail of RAGE that binds with SLP76, we generated various mutants of RAGE for transfection into HEK293 cells and found that the DCT region but not the D-domain-like docking site was the key structure mediating the interaction of RAGE with SLP76 (Fig. 2b). Bioinformatic analysis showed that the cytosolic tail of RAGE contains 4 potential phosphorylation sites (Y^391^, Y^399^, Y^400^, and T^401^). To clarify whether these phosphorylation sites are critical for RAGE-mediated signaling, we constructed the corresponding phosphorylation site mutants and found that mutation of these phosphorylation sites did not significantly influence the signaling processes induced by AGEs (Supplementary Fig. 6).

In search for the specific domain of SLP76 that binds with RAGE, different mutants of SLP76 were generated for overexpression and immunoprecipitation. Interestingly, the SAM domain was indispensable for the interaction of SLP76 with the cytosolic tail of RAGE (Fig. 2c) and subsequent AGE-induced signal transduction (Fig. 4h). Further experiments with mutants of tyrosine sites near the SAM domain demonstrated that these phosphorylation sites (Y^113^, Y^128^, and Y^145^), did not significantly influence AGE-induced activation of signaling pathways in macrophages (Fig. 4h), although the Y^113^ site slightly influenced the interaction between RAGE and SLP76 (Fig. 2c). Our results support the important concept that the association of SLP76 with the cytosolic tail of RAGE via the SAM domain mediates downstream signal transduction. SAM domains, which feature a high content of α-helices, are found in all subcellular compartments and participate in a wide variety of cellular processes, including signal transduction, protein–protein interactions, gene transcription regulation, and protein translational control^47–49^. Considering the indispensable role of the SAM domain of SLP76 in RAGE-mediated signaling, targeting the SAM domain may provide a therapeutic strategy for diseases caused by RAGE overactivation.

As an in vivo ligand for RAGE, HMGB1 is essential to the inflammatory response of sepsis. A long-lasting elevation in circulating HMGB1 levels that correlated with in-hospital mortality was identified in septic patients^50,51^. Furthermore, administration of an anti-HMGB1 neutralizing antibody to mice subjected to CLP led to a higher survival rate^52^. Thus, the beneficial effect of RAGE inhibition in sepsis is at least partially attributed to inhibition of one of its ligands, HMGB1. In the CLP mouse model, the concentration dynamics of AGEs and HMGB1 in the blood differed; AGEs increased in the early stage of sepsis and peaked at 3 h after CLP, while HMGB1 accumulated in the blood over time (Fig. 6b, c).

To evaluate the potential clinical application of targeting SLP76, we used a CPP to deliver the SAM domain into cells to disrupt the interaction of SLP76 with RAGE via competitive blocking the binding of endogenous SLP76 to the cytosolic tail of RAGE. As expected, pretreatment with the TAT-SAM synthetic peptide significantly suppressed AGE-induced activation of signaling pathways, resulting in a decrease in the production of proinflammatory mediators in macrophages. Furthermore, administration of the TAT-SAM peptide by tail vein injection to mice 1 h post CLP not only inhibited cytokine release but also decreased the concentration of HMGB1 in the blood (Fig. 6f). Consequently, tissue damage was significantly reduced, and the survival of mice was prolonged (Fig. 6).

**Fig. 7:**
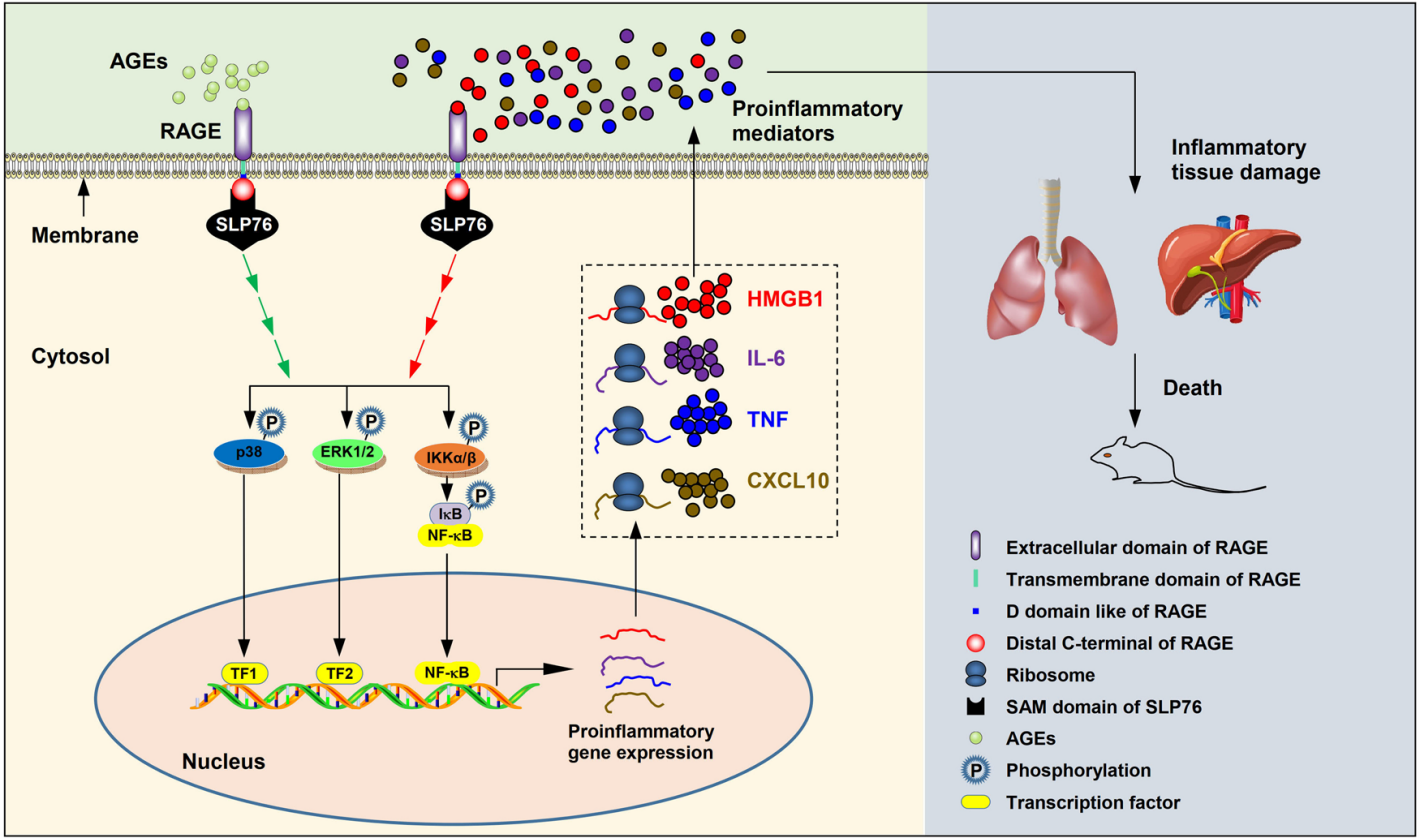
Pivotal function of SLP76 in RAGE-mediated intracellular signaling in sepsis. At the onset of sepsis, AGEs are rapidly released and accumulate in the blood. After binding of AGEs with the cell surface receptor RAGE, SLP76 is recruited to the cytosolic tail of RAGE as an adaptor protein to transduce signals from the membrane into the cell, resulting in rapid activation of the p38 MAPK, Erk1/2, and IKKα/β pathways via sequential protein phosphorylation. Activation of downstream kinases results in phosphorylation of transcription factors (TFs) such as NF-*κ*B, thus enhancing the gene transcription of proinflammatory mediators, including TNF, IL-6, CXCL10, HMGB1, etc. The DAMP molecule HMGB1 acts on RAGE, initiating the second wave of protein kinase cascade activation, which generating a positive feedback mechanism to maintain and amplify the proinflammatory response. Overproduction of proinflammatory factors causes tissue damage and finally leads to multiple organ failure and death in mice.

Based on the above findings, we concluded that the interaction of RAGE with its ligands recruits SLP76 to the cytosolic tail of RAGE, resulting in rapid activation of downstream signaling pathways, thus initiating gene transcription of proinflammatory mediators, including TNF, IL-6, CXCL10, HMGB1, etc. The upregulated HMGB1 binds to RAGE, which triggers and amplifies the inflammatory response. Through this positive feedback mechanism, large amounts of proinflammatory factors are produced, causing a persistent inflammatory response and severe tissue damage, finally leading to multiple organ failure and death in mice (Fig. 7). It should be noted that RAGE has functions that go beyond myeloid and lymphoid cells, such as endothelial cells, which lack of the SLP76 expression^53^. On the other hand, SLP76 has been implicated as a signaling component of T-cell receptor (TCR), which functions as a scaffold protein to transduce signals^54^. All these studies indicate that despite the binding partnership of RAGE and SLP76 in myeloid cells, the functions of RAGE and SLP76 can also be executed independently in other cells.

Here in the present study, our finding that the interaction of SLP76 with RAGE is mediated by the SAM domain not only sheds light on understanding the mechanism of RAGE-directed signal transduction but also provides a potential therapeutic target for sepsis. More prospectively, our findings on RAGE signaling may open a window to understanding other RAGE-associated diseases, such as DM, CVDs, and neurodegenerative diseases.

However, our study has some limitations. First, the composition of the SLP76 adaptor complex has not been identified; thus, the gap in knowledge about the relationship between RAGE and downstream kinase cascades is not completely filled. Second, in this study, the therapeutic effect of TAT-SAM on sepsis was observed only in mice; clinical trials are needed to assess the effect and safety of this strategy targeting the SAM domain of SLP76. Through comprehensive efforts, a technique for therapeutic interventions for sepsis could be developed.

## Methods

### Human blood samples

Blood samples were collected from patients admitted to the surgical ICU in the Department of Intensive Care Medicine, General Hospital of Southern Theatre Command of PLA, Guangzhou, China. Blood storage and usage for research were approved by all participants with signing written informed consent. The experiments performed in this study were approved and guided by the ethical committee of General Hospital of Southern Theatre Command of PLA. Sepsis was diagnosed according to The Third International Consensus Definitions for Sepsis and Septic Shock (Sepsis-3)^2^.

### Mice and cell lines

All C57BL/6 mice used in the experiments were male and 8–12 weeks old. WT C57BL/6 mice were purchased from Jackson Laboratory (Bar Harbor, ME, USA). All transgenic mice were of the C57BL/6 strain. *SLP76*^*−/−*^ mice were generated by Cyagen Biosciences Inc. (Guangzhou, China). *AGER*^*−/−*^ mice were acquired from Kanazawa University (Kanazawa, Japan). All the mice were kept in specific pathogen-free conditions where temperature, humidity, dark/light cycle of 12 h/12 h, and air exchange rate of 12–15 times per hour are maintained at a constant level. RAW264.7 cells (TIB-71) and HEK293 cells (CRL-1573) were obtained from the American Type Culture Collection (ATCC; Manassas, VA, USA) and maintained in Dulbecco’s modified Eagle’s medium (DMEM) supplemented with 10% fetal bovine serum (FBS), 100 U/ml penicillin, and 0.1 mg/ml streptomycin at 37 °C in a 5% CO_2_ incubator. All animal experiments were approved by the Southern Medical University Animal Care and Use Committee (Southern Medical University, Guangzhou, China).

### Reagents and antibodies

AGEs-BSA (#2221-10) was purchased from Biovision (Milpitas, CA, USA). The recombinant mouse HMGB1 was purchased from Abcam (#ab181949). Antibodies against ERK1/2 (#9102), phospho-ERK1/2 (Thr202/Tyr204) (#9101), SAPK/JNK (#9252), phospho-SAPK/JNK (Thr183/Tyr185) (#9251), p38 MAPK (#9212), and phospho-p38 MAPK (Thr180/Tyr182) (#9211) and antibodies against phospho-IKKα/β (Ser176/180) (#2679), SLP76 (#4958), and the HA tag (C29F4) (#3724) were purchased from Cell Signaling Technology (Boston, MA, USA). Polyclonal antibodies against RAGE (N-16) (#sc-8230) and IKKα/β (H-470) (#sc-7607) were obtained from Santa Cruz Biotechnology (Dallas, TX, USA). Anti-Flag tag (#F3165) monoclonal antibodies, ANTI-FLAG^®^ M2 affinity gel (#A2220) and EZview^TM^ Red anti-HA affinity gel (#E6779) were obtained from Sigma (St. Louis, MO, USA). siRNAs targeting SLP76 (#sc-36502) and RAGE (#sc-36375) and the corresponding control siRNAs (#sc-142627) were obtained from Santa Cruz Biotechnology. Protein G beads (#11719416001) were obtained from Roche (Mannheim, Germany), and a Plasmid Plus Midi Kit (#12945) for DNA preparation was purchased from Qiagen (Hilden, Germany). TAT and TAT-SAM peptides were synthesized by Bootech BioScience & Technology (Shanghai, China).

### T7 phage display system

A T7Select^®^ human lung complementary DNA (cDNA) library (#70646-3) containing the BLT5615 bacterial host was purchased from Novagen (Madison, WI, USA). An aliquot (2.0 × 10^10^ pfu) of the T7 phage library was incubated with GST-RAGE(CT) at 4 °C overnight. Nonspecifically bound bacteriophages were removed by washing five times with 200 μl of Tris-HCl (pH 8.0) containing 60 mM NaCl and 0.3% Tween 20, and the remaining bacteriophages specifically binding with the cytosolic tail of RAGE were rescued with elution buffer. Following three rounds of screening, host *E. coli* BLT5615 cells were infected with bacteriophage-containing supernatant at 37 °C and cultured to an OD_600_ of ~0.5. Then, the mixture was seeded on Luria-Bertani (LB) plates. Plaques were counted for bacteriophage titration and randomly picked for bacteriophage expansion. Bacteriophages were lysed in extraction buffer (100 mM NaCl, 6 mM MgSO_4_ in 20 mM Tris-HCl, pH 8.0) for preparation and subsequent sequencing of bacteriophage DNA. The procedures were repeated for three rounds until the desired enrichment was obtained. The enrichment ratio was defined as the pfu of recovered bacteriophage divided by the pfu of input bacteriophage.

### Construction of expression plasmids

Prokaryotic expression plasmids for GST-RAGE(CT) and His-SLP76 were constructed by using the corresponding primers (Supplementary Table 2) according to a routine molecular cloning method^55^. Eukaryotic expression plasmids for Flag-tagged WT SLP76 or its mutants were constructed with the pcDNA3 vector as previously reported. Similarly, expression plasmids for HA-tagged RAGE and its mutants were constructed with the pcDNA3 vector^56^. The ECFP-fused RAGE expression plasmid was constructed with pECFP-N1, and the expression plasmids for EYFP-fused SLP76 and its SAM domain-deficient mutant were constructed with pYCFP-N1^57^. Specific primers used to generate these constructs and enzyme sites used for digestion are listed in Supplementary Table 2. All constructed plasmids were sequenced, and the correct clones were selected for the next experiments.

### In vitro binding assay of cytosolic tail of RAGE and SLP76

The plasmid pGEX-KG-RAGE(CT) was transformed into the *E. coli* BL21 strain to produce the GST-RAGE(CT) fusion protein, and the plasmid pET-14b-SLP76 was transformed into the *E. coli* BL21(DE_3_) strain to produce the His-SLP76 fusion protein. The GST-RAGE(CT) fusion protein was purified with GST-binding resin (Novagen, Madison, WI, USA) and eluted with reduced glutathione. The His-SLP76 fusion protein was purified with Ni^2+^-NTA resin (Qiagen, Hilden, Germany) and eluted with elution buffer [50 mM NaH_2_PO_4_, 300 mM NaCl, and 250 mM imidazole (pH 8.0)]. After incubation with the GST-RAGE(CT) fusion protein or GST, His-SLP76 was pulled down with Ni^2+^-NTA beads.

### SDS-PAGE

Purified proteins or precipitates from the pulldown experiments were resolved by sodium dodecyl sulfate-polyacrylamide gel electrophoresis (SDS-PAGE) followed by routine staining with Coomassie brilliant blue.

### Transient transfection of DNA and siRNA

HEK293 cells (3 × 10^5^) were cultured to a confluence of 80–90% in a 60 mm dish. Then, cells were transfected with 2 μg of total DNA by using 6 μl GeneJammer reagent (#204130, Agilent, Santa Clara, CA, USA) mixed with Opti-MEM (200 μl final volume) and were then incubated for 5 min at room temperature. RAW264.7 cells (1 × 10^5^) were transfected with control siRNA, siRNA targeting *AGER* or siRNA targeting *SLP76* using HiPerFect reagent (#301705, Qiagen, Hilden, Germany). SiRNA diluted in HiPerFect transfection reagent was premixed with Opti-MEM medium at room temperature for 5 min and incubated with cells in a 24-well plate for 48 h.

### Co-IP

After transfection for 24 h, HEK293 cells were treated with AGEs at the indicated times and doses. Then, cells were collected and lysed with lysis buffer, and protein was quantitated with a bicinchoninic acid (BCA) kit (#23227, Thermo Fisher Scientific, Waltham, MA, USA). Total input protein (500 µg) was incubated with anti-FLAG M2 beads or anti-HA affinity gel overnight at 4 °C. After brief centrifugation, samples were washed five times with lysis buffer containing 0.1% Tween 20. Samples were then resolved by SDS-PAGE and subjected to western blotting with a specific antibody against the HA or Flag tag.

Cultured RAW264.7 cells were treated with AGEs as indicated. Then, cells were harvested and lysed on ice. Protein was quantitated with a BCA kit, and 500 µg of input protein was incubated with 1 µg of anti-RAGE antibody and 20 µl of protein G-Sepharose beads (Roche, Mannheim, Germany) overnight at 4 °C. Immunoprecipitation was performed as described above and was followed by western blotting with a specific antibody against RAGE or SLP76.

### Western blot analysis

Cultured cells in 6-well plates were harvested, washed twice with PBS, and lysed for 30 min on ice in 150 μl of cell lysis buffer (#9803) from CST (Boston, MA, USA) containing 1 mM PMSF and 2 μg/ml leupeptin. Proteins were quantified with the BCA method. Protein samples were resolved by 10% SDS-PAGE, transferred to a nitrocellulose (NC) membrane (#GE10600002), and blocked for 1 h with 5% nonfat milk in Tris-buffered saline containing 0.1% Tween 20 (TBST) at room temperature. After incubation with a specific primary antibody overnight at 4 °C, the membrane was washed with TBST three times. Then, the membrane was incubated with a secondary antibody for 1 h at room temperature. Enhanced chemiluminescence (ECL) (#32109, Thermo Fisher Scientific, Waltham, MA, USA) was used to visualize the protein bands, and images were acquired in a ChemiDoc™ Imaging System (Bio-Rad, Hercules, CA, USA). Each experiment was repeated at least three times.

### FRET measurement

HEK293 cells were cotransfected with pECFP-RAGE and pEYFP-SLP76 or pEYFP-SLP76(ΔSAM) using GeneJammer reagent (Agilent, Santa Clara, CA, USA). Live cells were visualized 36 h after transfection by using an inverted fluorescence microscope (Zeiss Axiovert 200 M). For FRET measurement, cells were observed with excitation and emission bandpass filters of 420 nm to 450 nm (Chroma Exciter 436/25) and 520–550 nm (Chroma Emitter 535/30), respectively. To correct for fluorescence bleedthrough into the FRET channel, cells transfected with either pECFP-RAGE and pEYFP-SLP76 or pEYFP-SLP76(ΔSAM) alone were used to determine the donor or acceptor correction factor. Images of CFP-RAGE and YFP-SLP76 or YFP-SLP76(ΔSAM) expressed in the cells were sequentially acquired in the donor (CFP) channel, acceptor (YFP) channel, and FRET channel under identical conditions. The image acquired in the FRET channel was evaluated using Carl Zeiss AxioVision FRET 4.6 software, and FRET values were calculated as described previously^57^.

### SPRi

To measure the binding affinity of the cytosolic tail of RAGE with the SAM domain of SLP76, a PlexArray HT A100 (Plexera, USA) was used to monitor the whole procedure in real time. Briefly, a chip with well-prepared biomolecular microarray was assembled with a plastic flow cell for sample loading. The synthesized peptide of RAGE cytosolic tail was prepared at 5 mM concentration in Tris-buffered saline (TBS) running buffer (20 mM Tris and 100 mM NaCl [pH 7.5]), and a 10 mM glycine-HCl buffer (pH 2.0) was used as regeneration buffer. The synthesized peptide of SLP76 SAM domain flowed at increasing concentrations (250, 500, 1000, 2000 nM). Binding data were collected and analyzed by a commercial SPRi analysis software (Plexera SPR Data Analysis Module, Plexera, USA).

### Isolation of BMDMs

BMDMs were isolated from WT, *AGER* knockout, and *SLP76* knockout mice. Mice were euthanized, and the femur and tibia were isolated and sterilized in 70% ethanol for 15 s and then washed with PBS. A 25-gauge needle was used to flush out the bone marrow with DMEM supplemented with 1% FBS. Bone marrow cells were passed through a cell strainer (70 μm), washed with PBS, and plated into a 10 cm^2^ dish at a density of 1 × 10^7^ cells/10 ml medium. BMDMs were grown in DMEM supplemented with 10% FBS and M-CSF (#130-094-129) (20 ng/ml) at 37 °C, and the medium was refreshed on day 3. After growth for 7 days, the cells were ready for use in subsequent experiments.

### ELISA

Serum was separated from patient samples for measurement of AGEs with an ELISA kit (#MBS267540, MyBioSource, San Diego, CA, USA) according to the manufacturer’s instructions. RAW264.7 cells were transfected with control siRNA, siRNA targeting *AGER,* or siRNA targeting *SLP76* for 48 h. Then, cultured cells were incubated with or without AGEs (100 μg/ml) for 12 h, and the supernatant was collected for the measurement of TNF (#MTA00B) and IL-6 (#M6000B) levels with ELISA kits (R&D Systems, Minneapolis, MN, USA) according to the manufacturer’s instructions.

### Cytokine measurement with the LiquiChip system

Cytokine levels in cultured cell supernatants were measured with the LiquiChip system, which uses bead-based xMAP (flexible multianalyte profiling) technology, in accordance with the manufacturer’s instructions^58–61^.

### Quantitative real-time PCR

Total RNA was isolated from cells with TRIzol reagent (#15596026, Invitrogen, Carlsbad, California, USA). The RNA concentration was determined by using a NanoDrop spectrophotometer (Thermo Fisher Scientific, Waltham, MA, USA). Complementary DNA was reverse transcribed with PrimeScript RT Master Mix (#RR036A, Takara) according to the manufacturer’s protocol. Real-time PCR was performed with specific primers (Supplementary Table 3) in a 7500 Real-Time PCR System (Applied Biosystems, Foster City, CA). The reaction mixture was preheated for 10 min at 95 °C, followed by 45 cycles of denaturation at 95 °C for 30 s, annealing at 55 °C for 30 s, and extension at 72 °C for 30 s. Beta-actin was used as the internal control. The 2^−ΔΔCt^ method was employed to evaluate mRNA expression^62^.

### Animal model of CLP

C57BL/6 mice were anesthetized by isoflurane inhalation. A midline laparotomy was performed in a sterile field. The cecum was exposed and was ligated and punctured twice with an 18-gauge hypodermic needle. This procedure was followed by slight compression to release a small amount of intestinal contents from the holes. Then, the cecum was returned to the peritoneal cavity, and the abdominal skin was closed. In parallel, the mice in the sham group underwent the same procedure without ligation or puncture. The animals were resuscitated by subcutaneous administration of 1 ml of normal saline (NS) immediately after surgery.

### Pathological examination

Lung and liver tissues were fixed with 10% formalin and embedded in paraffin. The blocks were cut into 5-μm slices and stained with hematoxylin and eosin (H&E). Morphologic changes in lung and liver tissues were evaluated by light microscopy, documented by imaging, and evaluated by two investigators in a blinded manner. Lung injury was assessed based on pulmonary edema as determined by alveolar wall thickening with vascular congestion and interstitial and alveolar leukocyte infiltration.

### Survival study

Male C57BL/6 WT mice were randomly assigned to the sham, CLP, CLP+TAT, or CLP+TAT-SAM group. Mice were subjected to CLP surgery and excluded if they died during surgery. The mice in the CLP+TAT, CLP+TAT-SAM, and CLP groups were administered the TAT peptide, TAT-SAM, or normal saline via tail vein injection 1 h after CLP. The mice in the sham group were subjected to surgery without CLP. The survival of mice was monitored for up to 7 days.

### Statistical analysis

The density of immunoblot bands was analyzed with ImageJ (NIH) software (Ver. 1.52a, http://imagej.nih.gov/ij). Data are presented as the means ± standard deviations (SDs) from at least three individual experiments. Statistical differences between two groups were analyzed by two-tailed unpaired *t* test. Complex data sets were compared by one- or two-way ANOVA using Statistical Package for the Social Sciences software (SPSS, v22.0). The Kaplan–Meier method was used to generate survival curves. *P* < 0.05 was considered statistically significant.

## Supplementary information

Supplementary Information

## Data Availability

The authors declare that all data are available in the article, supplementary information, or from the corresponding author upon reasonable request. Source data are provided as a Source Data file.

## Source data

Source Data

## References

[1] Rudd KE (2020). Global, regional, and national sepsis incidence and mortality, 1990–2017: analysis for the global burden of disease study. Lancet.

[2] Singer M (2016). The third international consensus definitions for sepsis and septic shock (Sepsis-3). JAMA.

[3] Mayr FB, Yende S, Angus DC (2014). Epidemiology of severe sepsis. Virulence.

[4] Angus DC, van der Poll T (2013). Severe sepsis and septic shock. N. Engl. J. Med..

[5] Seymour CW (2017). Time to treatment and mortality during mandated emergency care for sepsis. N. Engl. J. Med..

[6] Ibrahim ZA, Armour CL, Phipps S, Sukkar MB (2013). RAGE and TLRs: relatives, friends or neighbours?. Mol. Immunol..

[7] Riedemann NC, Guo RF, Ward PA (2003). Novel strategies for the treatment of sepsis. Nat. Med..

[8] Eppensteiner J (2019). Damage- and pathogen-associated molecular patterns play differential roles in late mortality after critical illness. JCI Insight.

[9] Vlassara H, Uribarri J (2014). Advanced glycation end products (AGE) and diabetes: cause, effect, or both?. Curr. Diabetes Rep..

[10] Bodiga VL, Eda SR, Bodiga S (2014). Advanced glycation end products: role in pathology of diabetic cardiomyopathy. Heart Fail. Rev..

[11] Gill V, Kumar V, Singh K, Kumar A, Kim JJ (2019). Advanced glycation end products (AGEs) may be a striking link between modern diet and health. Biomolecules.

[12] Lue LF (2001). Involvement of microglial receptor for advanced glycation endproducts (RAGE) in Alzheimer’s disease: identification of a cellular activation mechanism. Exp. Neurol..

[13] Andrades ME (2012). Plasma glycation levels are associated with severity in sepsis. Eur. J. Clin. Invest..

[14] Baumann M (2009). Advanced glycation endproducts in sepsis and mechanical ventilation: extra or leading man?. Crit. Care.

[15] Rai V (2012). Signal transduction in receptor for advanced glycation end products (RAGE): solution structure of C-terminal rage (ctRAGE) and its binding to mDia1. J. Biol. Chem..

[16] Bongarzone S, Savickas V, Luzi F, Gee AD (2017). Targeting the receptor for advanced glycation endproducts (RAGE): a medicinal chemistry perspective. J. Med. Chem..

[17] Park H, Adsit FG, Boyington JC (2010). The 1.5 Å crystal structure of human receptor for advanced glycation endproducts (RAGE) ectodomains reveals unique features determining ligand binding. J. Biol. Chem..

[18] Bopp C (2008). sRAGE is elevated in septic patients and associated with patients outcome. J. Surg. Res..

[19] Jones TK (2020). Plasma sRAGE acts as a genetically regulated causal intermediate in sepsis-associated acute respiratory distress syndrome. Am. J. Respir. Crit. Care Med..

[20] Hudson BI (2008). Interaction of the RAGE cytoplasmic domain with diaphanous-1 is required for ligand-stimulated cellular migration through activation of Rac1 and Cdc42. J. Biol. Chem..

[21] Zhao X, Liao YN, Huang Q (2018). The impact of RAGE inhibition in animal models of bacterial sepsis: a systematic review and meta-analysis. J. Int. Med. Res..

[22] Hudson BI, Lippman ME (2018). Targeting RAGE signaling in inflammatory disease. Annu. Rev. Med..

[23] Schmidt AM, Yan SD, Yan SF, Stern DM (2000). The biology of the receptor for advanced glycation end products and its ligands. Biochim. Biophys. Acta.

[24] Lutterloh EC, Opal SM (2007). Antibodies against RAGE in sepsis and inflammation: implications for therapy. Expert Opin. Pharmacother..

[25] Patel SG (2019). Cell-penetrating peptide sequence and modification dependent uptake and subcellular distribution of green florescent protein in different cell lines. Sci. Rep..

[26] Koren E, Apte A, Sawant RR, Grunwald J, Torchilin VP (2011). Cell-penetrating TAT peptide in drug delivery systems: proteolytic stability requirements. Drug Deliv..

[27] Kokkola R (2005). RAGE is the major receptor for the proinflammatory activity of HMGB1 in rodent macrophages. Scand. J. Immunol..

[28] Wang H, Yang H, Czura CJ, Sama AE, Tracey KJ (2001). HMGB1 as a late mediator of lethal systemic inflammation. Am. J. Respir. Crit. Care Med..

[29] Polat G, Ugan RA, Cadirci E, Halici Z (2017). Sepsis and septic shock: current treatment strategies and new approaches. Eurasia. J. Med..

[30] Fink MP, Warren HS (2014). Strategies to improve drug development for sepsis. Nat. Rev. Drug Discov..

[31] van Zoelen MA, Achouiti A, van der Poll T (2011). The role of receptor for advanced glycation endproducts (RAGE) in infection. Crit. Care.

[32] van Zoelen MA, van der Poll T (2008). Targeting RAGE in sepsis. Crit. Care.

[33] Feuerstein GZ (2008). Cardiac RAGE in sepsis: call TOLL free for anti-RAGE. Circ. Res..

[34] Liliensiek B (2004). Receptor for advanced glycation end products (RAGE) regulates sepsis but not the adaptive immune response. J. Clin. Invest..

[35] Lutterloh EC (2007). Inhibition of the RAGE products increases survival in experimental models of severe sepsis and systemic infection. Crit. Care.

[36] Juranek JK (2016). Soluble RAGE Treatment delays progression of amyotrophic lateral sclerosis in SOD1 mice. Front. Cell. Neurosci..

[37] Calfee CS (2008). Plasma receptor for advanced glycation end products and clinical outcomes in acute lung injury. Thorax.

[38] Hudson BI (2008). Identification, classification, and expression of RAGE gene splice variants. FASEB J..

[39] Ishihara K, Tsutsumi K, Kawane S, Nakajima M, Kasaoka T (2003). The receptor for advanced glycation end-products (RAGE) directly binds to ERK by a D-domain-like docking site. FEBS Lett..

[40] Thiyagarajan S., Leclerc E., Vetter S. The cytoplasmic domain of RAGE (Receptor for Advanced Glycation Endproduct) is selfsufficient to contribute cell adhesion in the presence of extracellular matrix. *FASEB J.* **33**, 680-5 (2019).

[41] Chuah YK, Basir R, Talib H, Tie TH, Nordin N (2013). Receptor for advanced glycation end products and its involvement in inflammatory diseases. Int. J. Inflam..

[42] Bezman NA (2008). Requirements of SLP76 tyrosines in ITAM and integrin receptor signaling and in platelet function in vivo. J. Exp. Med..

[43] Bounab Y (2014). Proteomic analysis of the SH2 domain-containing leukocyte protein of 76 kDa (SLP76) interactome in resting and activated primary mast cells. Mol. Cell. Proteom..

[44] Block H (2012). Crucial role of SLP-76 and ADAP for neutrophil recruitment in mouse kidney ischemia-reperfusion injury. J. Exp. Med..

[45] Fang N, Motto DG, Ross SE, Koretzky GA (1996). Tyrosines 113, 128, and 145 of SLP-76 are required for optimal augmentation of NFAT promoter activity. J. Immunol..

[46] Lewis JB (2018). ADAP is an upstream regulator that precedes SLP-76 at sites of TCR engagement and stabilizes signaling microclusters. J. Cell Sci..

[47] Schultz J, Ponting CP, Hofmann K, Bork P (1997). SAM as a protein interaction domain involved in developmental regulation. Protein Sci..

[48] Qiao, F. & Bowie, J. U. The many faces of SAM. *Sci. STKE* **2005**, re7 (2005). 10.1126/stke.2862005re7 15928333

[49] Vincenzi M, Mercurio FA, Leone M (2020). Sam domains in multiple diseases. Curr. Med. Chem..

[50] Yagmur E (2018). High mobility group box 1 as a biomarker in critically ill patients. J. Clin. Lab Anal..

[51] Valdes-Ferrer SI (2016). HMGB1 mediates anemia of inflammation in murine sepsis survivors. Mol. Med..

[52] Stevens NE (2017). Therapeutic targeting of HMGB1 during experimental sepsis modulates the inflammatory cytokine profile to one associated with improved clinical outcomes. Sci. Rep..

[53] Pollreisz A (2010). Receptor for advanced glycation endproducts mediates pro-atherogenic responses to periodontal infection in vascular endothelial cells. Atherosclerosis.

[54] Shim EK, Jung SH, Lee JR (2011). Role of two adaptor molecules SLP-76 and LAT in the PI3K signaling pathway in activated T cells. J. Immunol..

[55] Xu J (2014). Macrophage endocytosis of high-mobility group box 1 triggers pyroptosis. Cell Death Differ..

[56] Zhang YJ (2019). Axin-1 binds to Caveolin-1 to regulate the LPS-induced inflammatory response in AT-I cells. Biochem. Biophys. Res. Commun..

[57] Zhong TY (2009). Using FRET to study the interaction domain of TLR4 binding to MD-2 in living cells. Prog. Biochem. Biophys..

[58] Xu J (2005). Detection of severe acute respiratory syndrome coronavirus in the brain: potential role of the chemokine mig in pathogenesis. Clin. Infect. Dis..

[59] Jiang Y (2005). Characterization of cytokine/chemokine profiles of severe acute respiratory syndrome. Am. J. Respir. Crit. Care Med..

[60] Gao K (2019). Exosomes derived from septic mouse serum modulate immune responses via exosome-associated cytokines. Front. Immunol..

[61] Wang J (2015). Injury-induced MRP8/MRP14 stimulates IP-10/CXCL10 in monocytes/macrophages. FASEB J..

[62] Rao X, Huang X, Zhou Z, Lin X (2013). An improvement of the 2^(-delta delta CT) method for quantitative real-time polymerase chain reaction data analysis. Biostat. Bioinforma. Biomath..

