# Supplementary material for: Targeting adaptor protein SLP76 of RAGE as a therapeutic approach for lethal sepsis
Source: Nat Commun. 2021 Jan 12;12:308. doi: 10.1038/s41467-020-20577-3 (PMC7804203; doi:10.1038/s41467-020-20577-3)
Supplement: Supplementary file 1 — Supplementary Information [file 41467_2020_20577_MOESM1_ESM.pdf]

SUPPLEMENTARY MATERIALS:

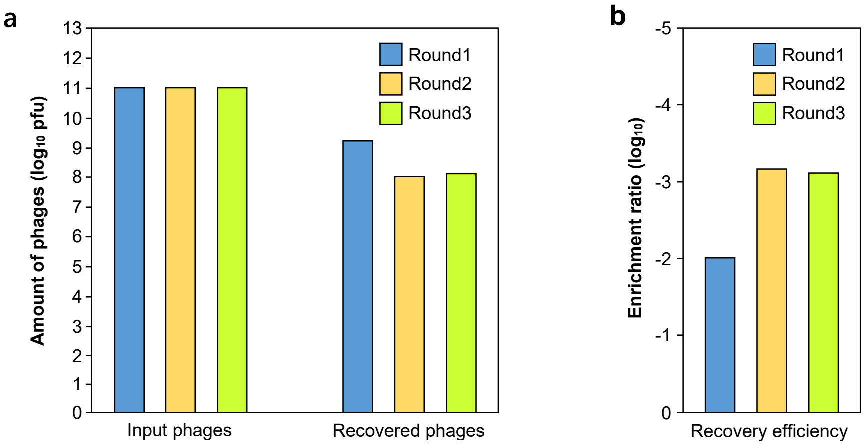

Supplementary Fig. 1 Biopanning to identify the binding partners of the cytosolic tail of RAGE.

a Input and recovered bacteriophages in rounds 1 to 3. The pfu of input bacteriophages in each round was 2.0×10^{11} pfu, and the pfu values of recovered bacteriophages in rounds 1 to 3 were 2.0×10^{9} pfu, 1.2×10^{8} pfu and 1.4×10^{8} pfu, respectively. **b** Enrichment ratios calculated for the biopanning process. The enrichment ratios for rounds 1 to 3 were 9.50×10^{-3}, 6.67×10^{-4} and 7.34×10^{-4}, respectively. Enrichment ratio = recovered bacteriophages/input bacteriophages. The result was acquired from a procedure of 3 round biopanning (*n*=1).

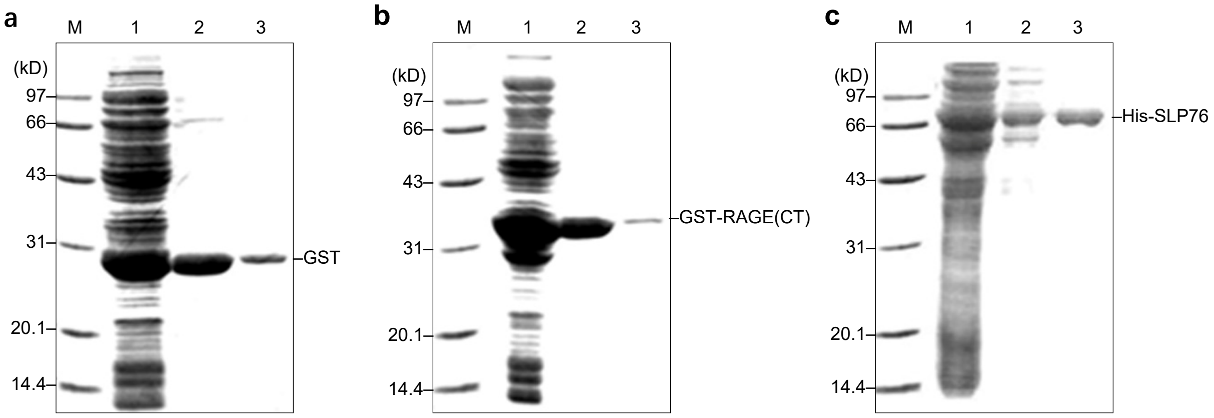

Supplementary Fig. 2 Purification of GST- or His-tagged recombinant proteins.

a Purification of GST protein. M, protein marker; lane 1, supernatant of *E. coli* BL21 carrying the GST expression plasmid pGEX-KG induced by IPTG; lanes 2 and 3, eluted GST protein. **b** Purification of the GST-fused cytoplasmic tail of RAGE, i.e., GST-RAGE(CT). M, protein marker; lane 1, supernatant of *E. coli* BL21 carrying the GST-RAGE(CT) expression plasmid pGEX-KG induced by IPTG; lanes 2 and 3, eluted GST-RAGE(CT) recombinant protein. **c** Purification of His-SLP76 recombinant protein (His-SLP76). M, protein marker; lane 1, supernatant of *E. coli* BL21(DE3) carrying the His-SLP76 expression plasmid induced by IPTG; lanes 2 and 3, eluted GST recombinant protein. All experiments were repeated three times with similar results and one representative experiment is shown.

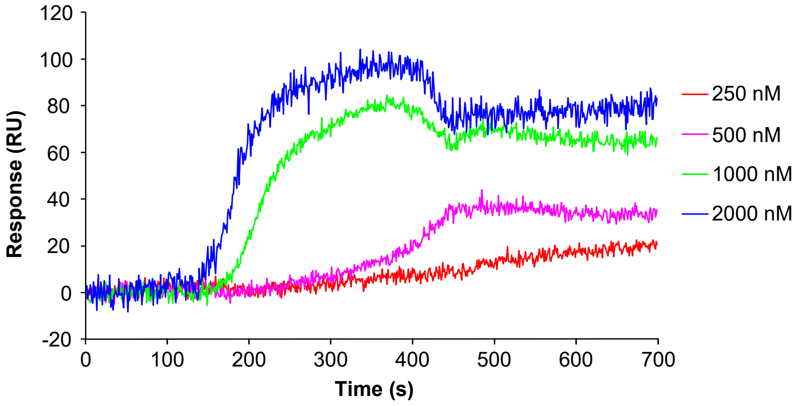

Supplement Fig. 3 SPRi assay for the binding affinity of RAGE and SLP76.

The synthesized peptide of RAGE cytosolic tail (5 μM) was immobilized in replicate (*n*=4) on the SPRi chip. The synthesized peptide of SLP76 SAM domain flowed at increasing concentrations (250 nM, 500 nM, 1000 nM, 2000 nM) and SPRi curves were recorded and analyzed by Plexera SPR Data Analysis Module.

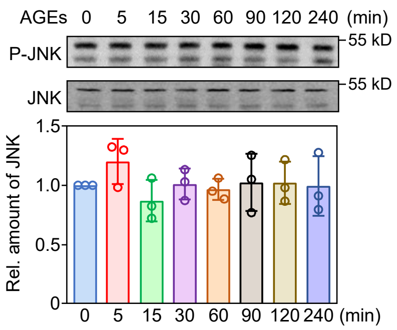

Supplementary Fig. 4 Effects of AGEs on the phosphorylation of JNKs in macrophages. RAW264.7 cells were incubated with AGEs (100 μg/ml) for different times. Cells were harvested for detection of JNK phosphorylation by Western blotting with a specific antibody against total or phosphorylated JNKs. Data were from three independent experiments (*n*=3) and are shown as the mean ± SD. There was no significant difference between groups (one-way ANOVA with Tukey post hoc). Source data are provided as a Source Data file.

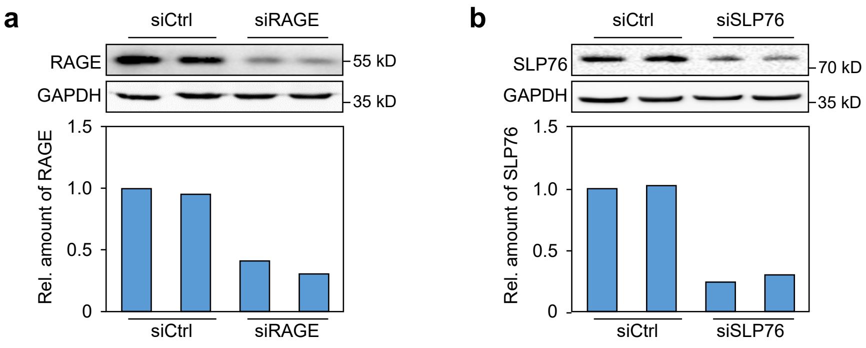

Supplementary Fig. 5 Efficiency of gene knockdown with specific siRNAs targeting *AGER* or *SLP76* in macrophages.

a Efficiency of gene knockdown in RAW264.7 cells by transfection of siRNA targeting *AGER*. **b** Efficiency of gene knockdown in RAW264.7 cells by transfection of siRNA targeting *SLP76*. The result was from an experiment (n=1) with two biological replicates. Source data are provided as a Source Data file.

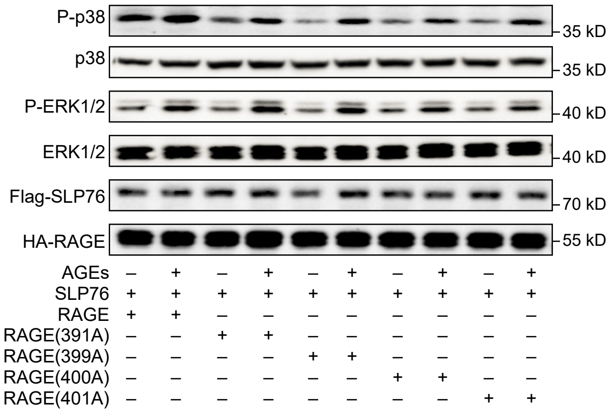

Supplementary Fig. 6 The potential phosphorylation sites of RAGE were not required for RAGE-mediated activation of proinflammatory signaling pathways.

HEK293 cells were cotransfected with plasmids expressing WT, SLP76 and RAGE or its phosphorylation site mutants for 24 h and were then treated with AGEs (100 μg/ml) for 15 min. Phosphorylation of p38 MAPK, ERK1/2 and IKKα/β was detected by Western blotting with specific antibodies (*n*=3). Source data are provided as a Source Data file.

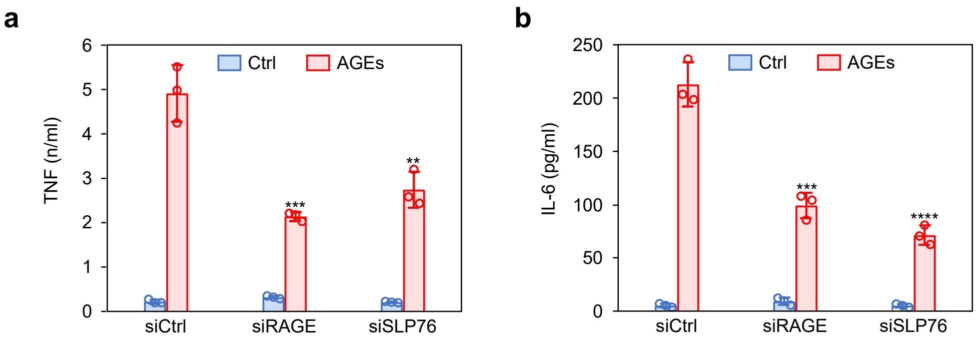

Supplementary Fig. 7 Effects of *AGER* or *SLP76* gene knockdown on cytokine production in macrophages induced by AGEs.

a Inhibition of *AGER* gene knockdown on AGE-induced cytokine production. RAW264.7 cells were transfected with control siRNA or specific siRNA targeting *AGER* for 48 h and were then stimulated with AGEs for 12 h. The levels of TNF and IL-6 in culture media were measured by ELISA. Data were from three independent experiments (n=3) and are shown as the mean ± SD. ** P<0.01, *** P<0.001 compared with the control siRNA group (one-way ANOVA with Tukey post hoc). **b** Inhibition of *SLP76* gene knockdown on AGE-induced cytokine production. RAW264.7 cells were transfected with control siRNA or specific siRNA targeting *SLP76* for 48 h and were then stimulated with AGEs for 12 h. The levels of TNF and IL-6 in culture media were measured by ELISA. Data were from three independent experiments (n=3) and are shown as the mean ± SD. *** P<0.001, **** P<0.0001 compared with the control siRNA group (one-way ANOVA with Tukey post hoc). Source data are provided as a Source Data file.

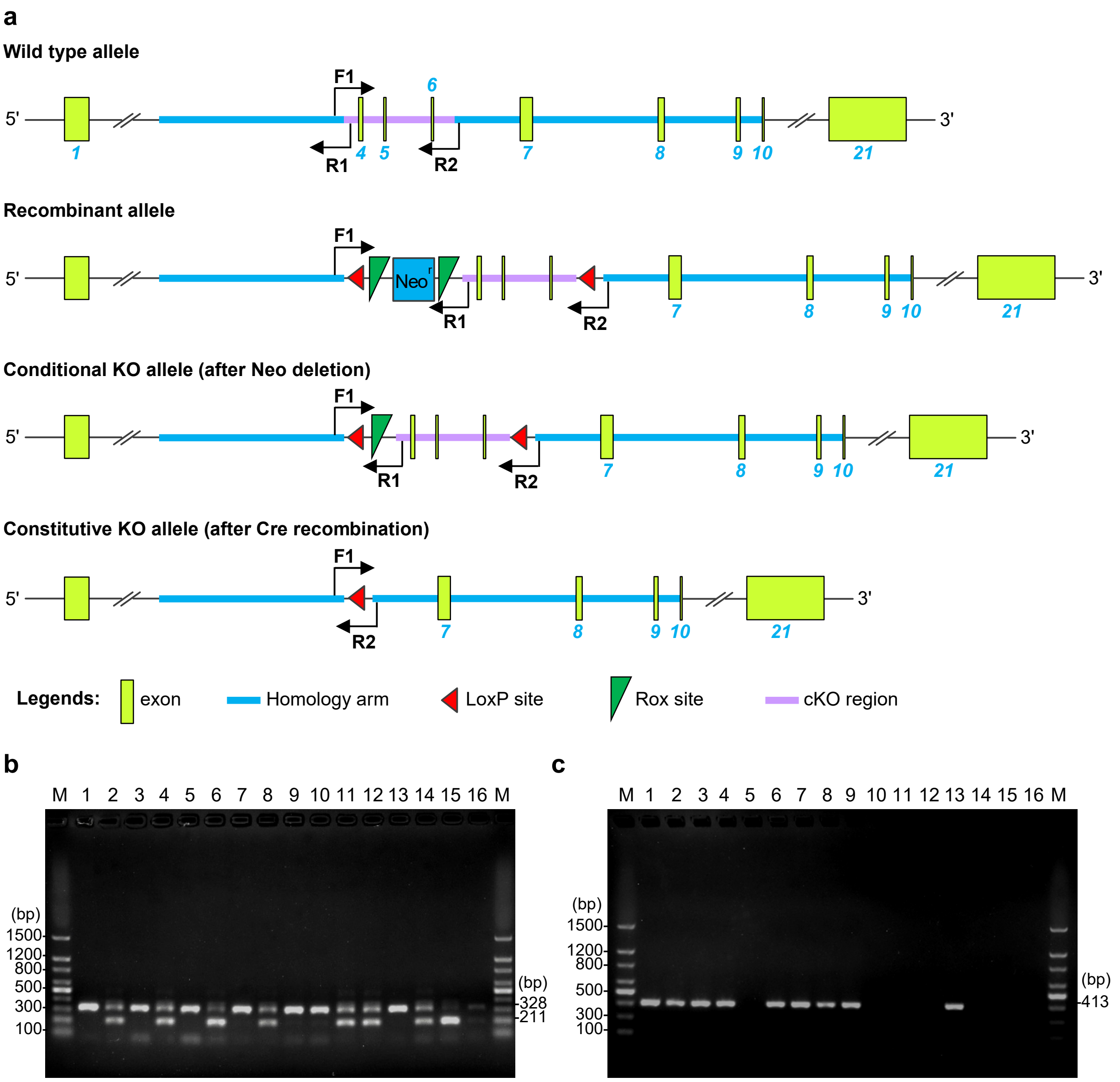

Supplementary Fig. 8 Generation and identification of mice with *SLP76* deletion in myeloid lineage cells.

a Schematic diagram of the establishment of *SLP76* conditional knockout. Mice with *SLP76* deletion in myeloid lineage cells were generated by crossing *SLP76*$^{loxP/loxP}$ mice with *Lyz2-Cre* knockin mice. **b** Identification of mice with the *loxP/loxP* genotype (PCR product length, 328 bp). The lengths of the PCR products from the WT and heterozygous mice were 211 bp and 413 bp, respectively. **c** Identification of mice with the *Lyz2-Cre*$^{+}$ genotype (PCR product length, 413 bp).

The genotype of mice was identified 4 weeks after birth, and the representative results are shown.

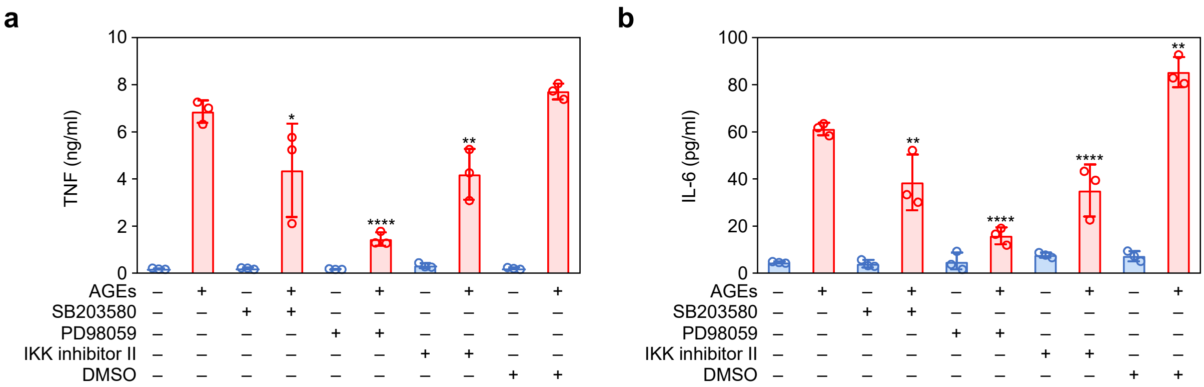

Supplementary Fig. 9 Suppression of AGE-induced cytokine production by specific inhibitors of p38 MAPK, ERK1/2 and IKKα/β.

a Suppression of AGE-induced TNF secretion after treatment with specific inhibitors of p38 MAPK, ERK1/2 and IKKα/β**.** RAW264.7 cells were pretreated with different inhibitors for 1 h and were then stimulated with AGEs (100 μg/ml) for 12 h. The level of TNF in the culture medium was measured by ELISA. Data were from three independent experiments (*n*=3) and are shown as the mean ± SD. * P<0.05, ** P<0.01, **** P<0.0001 compared with the AGE treatment group (one-way ANOVA with Tukey post hoc). DMSO was used as the vehicle control. **b** Suppression of AGE-induced IL-6 secretion after treatment with specific inhibitors of p38 MAPK, ERK1/2 and IKKα/β**.** RAW264.7 cells were pretreated with different inhibitors for 1 h and were then stimulated with AGEs (100 μg/ml) for 12 h. The level of IL-6 in the culture medium was measured by ELISA. Data were from three independent experiments (*n*=3) and are shown as the mean ± SD. ** P<0.01, **** P<0.0001 compared with the AGE treatment group (one-way ANOVA with Tukey post hoc). DMSO was used as the vehicle control. Source data are provided as a Source Data file.

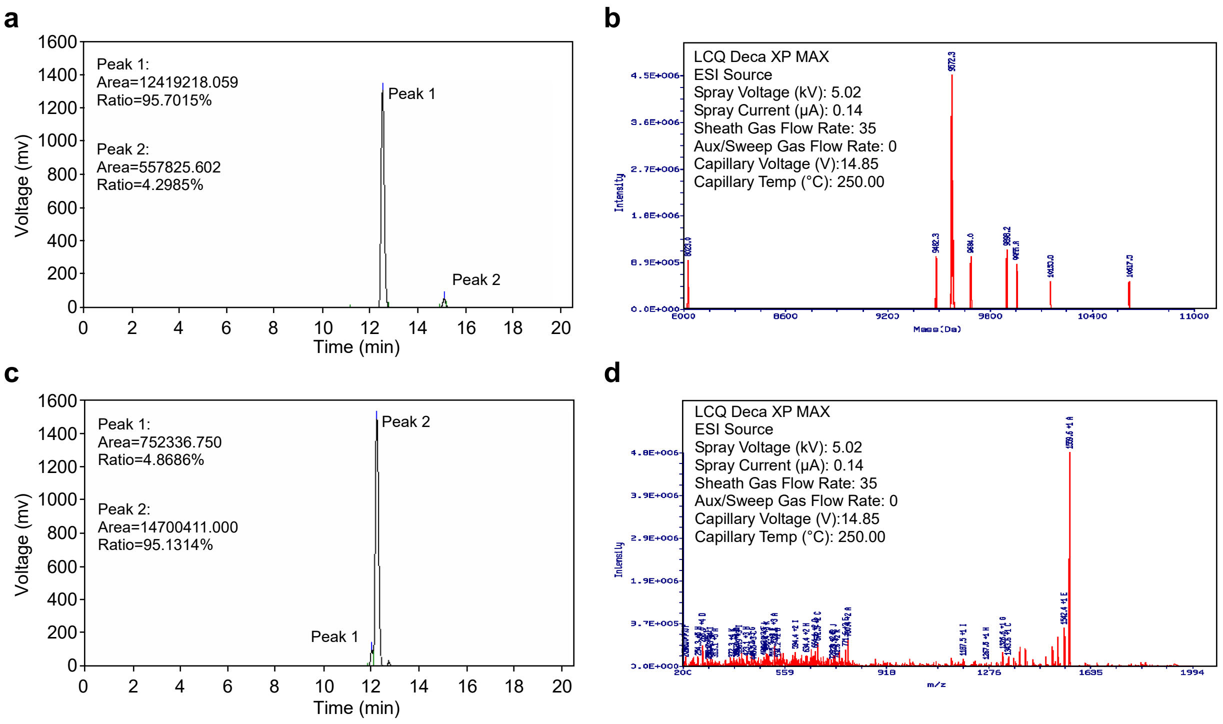

Supplementary Fig. 10 High-performance liquid chromatography and mass spectrometry analysis of the synthetic peptides.

a, b High-performance liquid chromatography (HPLC) (**a**) and mass spectrometry (**b**) were performed to determine the purity of the synthetic TAT-SAM peptide. The purity of the TAT-SAM peptide was 95.70%. **c, d** HPLC (**c**) and mass spectrometry (**d**) were performed to determine the purity of the synthetic TAT peptide. The purity of the TAT peptide was 95.13%.

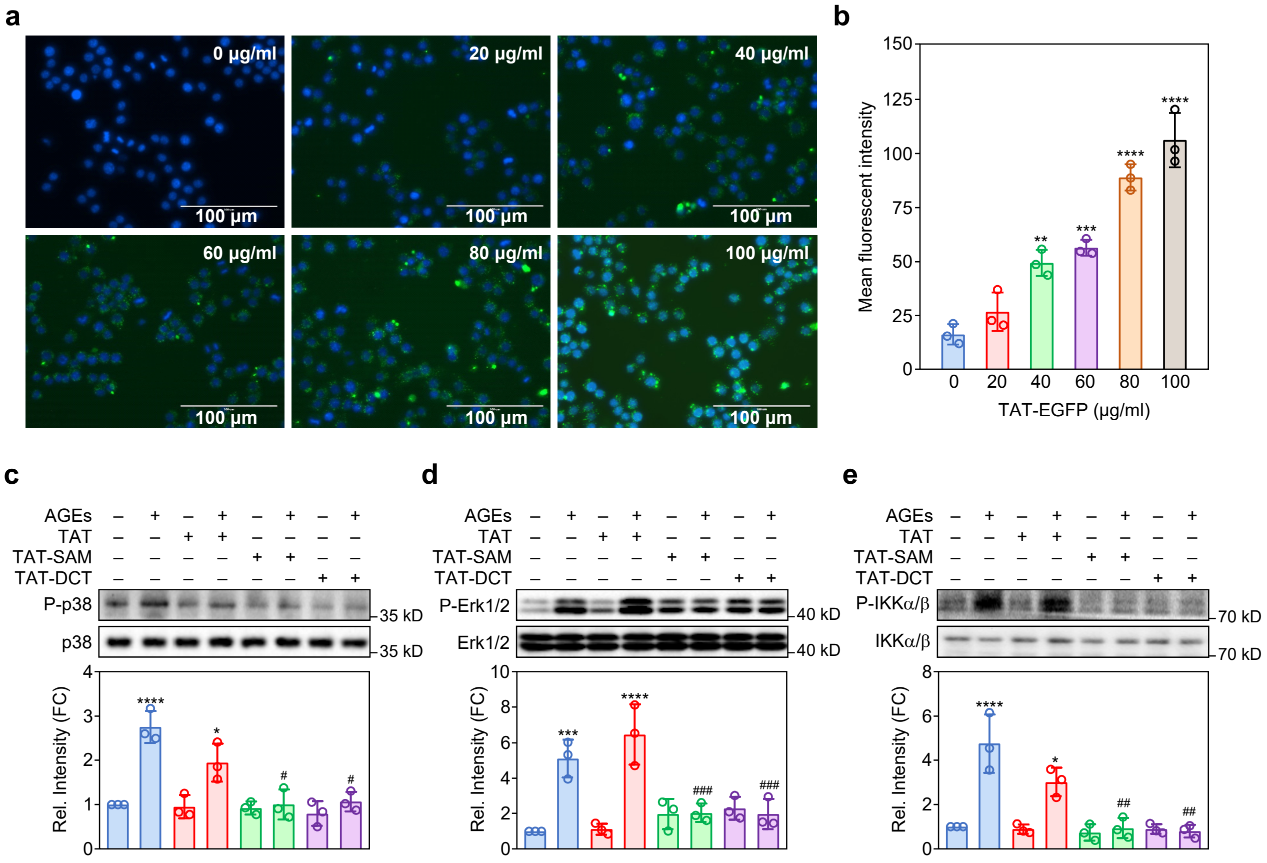

Supplementary Fig. 11 Inhibition of AGE-induced macrophage activation by the TAT-SAM peptide.

a Dose-dependent delivery of EGFP by the TAT CPP. RAW264.7 cells were incubated with TAT-fused EGFP recombinant protein (TAT-EGFP) at different doses for 1 h and observed by fluorescence microscopy (*n*=3). Scale bar: 100 μm. **b** Quantification of the mean fluorescence intensity in (**a**) by ImageJ software. Data were from three independent experiments (*n*=3) and are shown as the mean ± SD. ** *P*<0.01, *** *P*<0.001, **** *P*<0.0001 compared with the control (one-way ANOVA with Tukey post hoc). **c-e** Effects of TAT-fused peptides on AGE-induced activation of proinflammatory signaling pathways. RAW264.7 cells were pretreated with TAT, TAT-DCT or TAT-SAM for 1 h and were then stimulated with AGEs for 15 min. Activation of p38 MAPK (**c**), ERK1/2 (**d**) and IKKα/β (**e**) was detected by Western blotting. Data were from three independent

experiments (n=3) and are shown as the mean ± SD. * P<0.05, *** P<0.001, **** P<0.0001 compared with the untreated control group. $^{\#}$ P<0.05, $^{\#\#}$ P<0.01, $^{\#\#\#}$ P<0.001 compared with the TAT group (one-way ANOVA with Tukey post hoc). Source data are provided as a Source Data file.

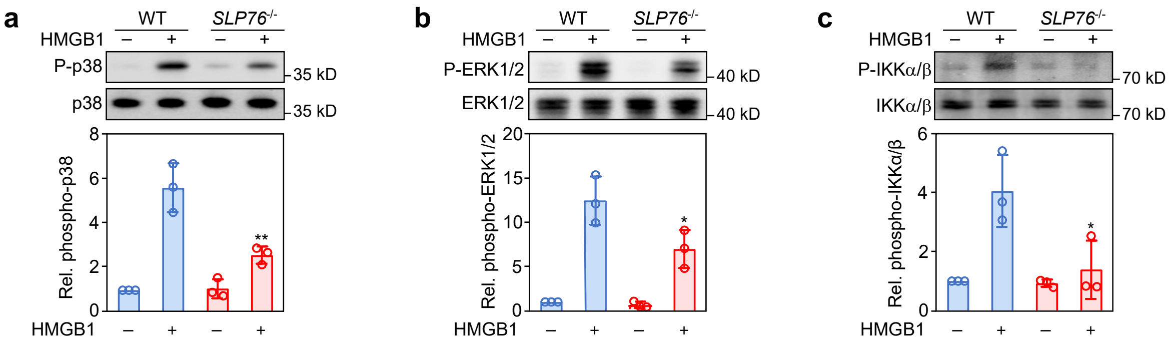

Supplementary Fig. 12 Effects of *SLP76* deficiency on phosphorylation of p38 MAPK, ERK1/2 and IKKα/β in BMDMs stimulated with HMGB1.

a-c BMDMs were isolated from WT or *SLP76*$^{-/-}$ mice and stimulated with or without HMGB1 (400 ng/ml) for 15 min. Western blotting was performed to detect phosphorylation of p38 MAPK, ERK1/2 and IKKα/β. Data were from three independent experiments (n=3) and are shown as the mean ± SD. * P<0.05, ** P<0.01 compared with the WT group (one-way ANOVA with Tukey post hoc). Source data are provided as a Source Data file.

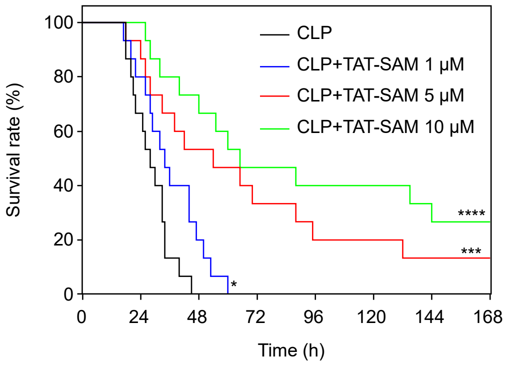

Supplementary Fig. 13 The dose-dependent effect of TAT-SAM on the survival of mice subjected to CLP modeling.

The mice were injected with different doses of TAT-SAM (1 μM, 5 μM, 10 μM) through tail vein 1 h after CLP (*n*=15). Administration of TAT-SAM peptide significantly improved the survival of CLP mice in a dose-dependent manner. Kaplan-Meier survival analysis was performed to evaluate the survival of septic mice. * P<0.05, *** P<0.001, **** P<0.0001 vs CLP group. Source data are provided as a Source Data file.

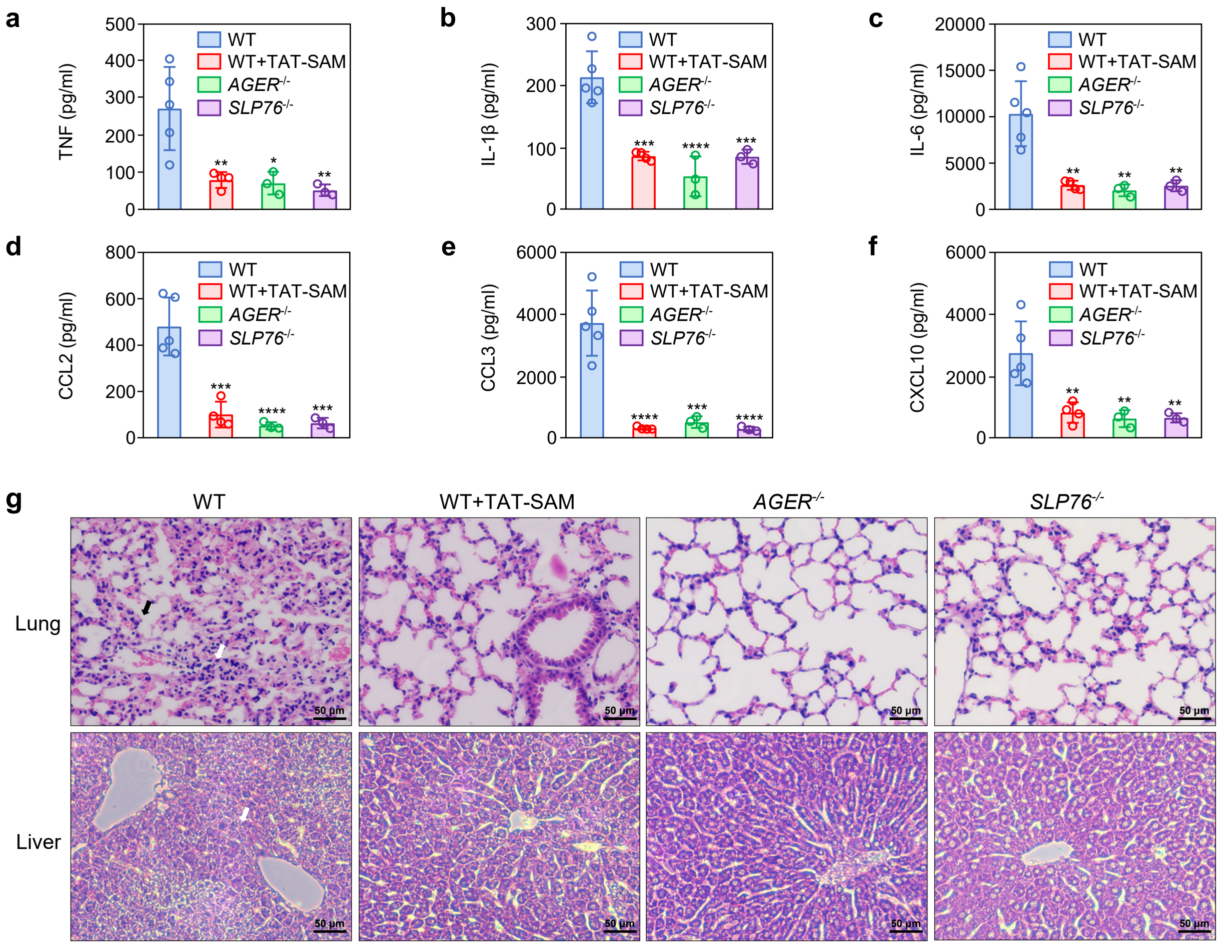

Supplementary Fig. 14 The effect of TAT-SAM peptide and *AGER* or *SLP76* gene deficiency on cytokine production and tissue damage.

a-f Inhibitory effect of the TAT-SAM peptide and gene deficiency of *AGER* or *SLP76* on the production of cytokines in the blood of septic mice. All the mice were subjected to CLP modeling, followed by serum collection 12 h after CLP (*n*=5 WT, *n*=4 TAT-SAM, *n*=3 $SLP76^{-/-}$ and *n*=3 $AGER^{-/-}$). The levels of blood cytokines were measured by using a multiplex LiquiChip system. Data were shown as the mean ± SD. * $P<0.05$, ** $P<0.01$, *** $P<0.001$, **** $P<0.0001$ compared with the WT group (one-way ANOVA with Tukey post hoc). **g** Protective effects of the TAT-SAM peptide and gene deficiency of *AGER* or *SLP76* on the damage of lung and liver tissues of septic mice. All the mice were subjected to CLP modeling. Twelve hours later, the lung and liver tissues

from mice were collected for pathological examination. H&E staining showed massive infiltration of inflammatory cells (white arrows), marked interalveolar septal thickening and alveolar edema (black arrows) in the lung tissues of septic mice. The pathology of liver tissues showed extensive liver damage and inflammatory cell infiltration (white arrows) in the mice subjected to CLP modeling (*n*=5 WT, *n*=4 TAT-SAM, *n*=3 *SLP76*$^{-/-}$ and *n*=3 *AGER*$^{-/-}$). Scale bar: 50 μm. Source data are provided as a Source Data file.

Supplementary Table 1. The potential binding proteins of RAGE cytosolic tail screened by T7 phage display.

Entry	Protein name	Gene name	GO-Biological process
Q13094	SH2 domain containing leukocyte specific phosphoprotein of 76 kD, (SLP76)	*LCP2*	Intracellular signal transduction; cytokine secretion; immune response
O60318	Germinal-center associated nuclear protein	*MCM3AP*	mRNA export from nucleus; nucleosome organization; poly(A)+ mRNA export from nucleus
Q8IWL2	Pulmonary surfactant-associated protein A1	*SFTPA1*	Cellular protein metabolic process; developmental process; opsonization
Q99460	26S proteasome non-ATPase regulatory subunit 1	*PSMD1*	Activation of MAPKK activity, innate immune response NIK/NF-kappaB signaling
Q03001	Dystonin	*DST*	Cell adhesion; cell motility; cytoplasmic microtubule organization
P15104	Glutamine synthetase	*GLUL*	Ammonia assimilation cycle; angiogenesis; cell population proliferation
Q8N554	Zinc finger protein 276	*ZNF276*	Transcription; transcription regulation
Q9UKY4	Protein O-mannosyl-transferase 2	*POMT2*	ER-associated misfolded protein catabolic process; positive regulation of protein O-linked glycosylation
Q9UK61	Protein TASOR	*TASOR*	Negative regulation of gene expression, epigenetic; negative regulation of single stranded viral RNA replication via double stranded DNA intermediate
Q86UU1	Pleckstrin homology-like domain family B member 1	*PHLDB1*	Positive regulation of basement membrane assembly involved in embryonic body morphogenesis; regulation of epithelial to mesenchymal transition
Q9Y4D7	Plexin-D1	*PLXND1*	Angiogenesis, endothelial cell migration; positive regulation of protein binding; synapse assembly

Supplementary Table 2. Information for the expression plasmids constructed in the study.

No.	Plasmid name	Tag	Cloning sites	Primer sequences
1	pGEX-KG-RAGE(CT)	GST	*Bam*H I<br>*Eco*R I	Forward: 5’-ATGGATCCCAAAGGCGGCAACGCCGAGGA-3’<br>Reverse: 5’-TGAATTCTCAAGGCCCTCCAGTACTACT-3’
2	pET-14b-SLP76	His	*Bam*H I<br>*Bam*H I	Forward: 5’-TAGGATCCGCACTGAGGAATGTGCCCTTTC-3’<br>Reverse: 5’-TAGGATCCCTATGGGTACCCTGCAGCATGC-3’
3	pcDNA3-Flag-SLP76	Flag	*Bam*H I<br>*Bam*H I	Forward: 5’-TAGGATCCGCACTGAGGAATGTGCCCTTTC-3’<br>Reverse: 5’-TAGGATCCCTATGGGTACCCTGCAGCATGC-3’
4	pcDNA3-Flag-SLP76(Y113A)	Flag	N/A	Forward: 5’-**GCT**GAAAGTCCCAATGATGACCAGG-3’<br>Reverse: 5’-ATCGTCTTCTTCAAAGGACGACCAGC-3’
5	pcDNA3-Flag-SLP76(Y128A)	Flag	N/A	Forward: 5’-**GCT**GAGTCCCCCAATGAGGAGGAAGAGGC-3’<br>Reverse: 5’-GTCTCCATCATCCTCCCCATCCTGGTCATCATTG-3’
6	pcDNA3-Flag-SLP76(Y145A)	Flag	N/A	Forward: 5’-**GCT**GAGCCGCCACCCTCCAATGACGAG-3’<br>Reverse: 5’-ATCCGCGTCATCTTCCACGGGTGCCTCTTC-3’
7	pcDNA3-Flag-SLP76(ΔSAM)	Flag	N/A	Forward: 5’-AGGAGCATCTTCACACGCAAACCCCAAGTCC-3’<br>Reverse: 5’-CTCTGAGCGAAAGGGCACATTCCTCAGTGCCAT-3’
8	pcDNA3-RAGE	N/A	*Kpn* I<br>*Eco*R I	Forward: 5’-ACGGTACCGCTGCCGGAACAGCAGTTGGAGC-3’<br>Reverse: 5’-ACGAATTCTCAAGGCCCTCCAGTACTACTCTCGC-3’
9	pcDNA3-HA-RAGE	HA	N/A	Forward: 5’-TTCCAGATTACGCTCAAAACATCACAGCCCGGAT-3’<br>Reverse: 5’-CATCGTATGGGTAAGCACCTACTACTGCCCCCCACA-3’
10	pcDNA3-HA-RAGE(S391A)	HA	N/A	Forward: 5’-**GCA**GAGGAACCTGAGGCAGGCGAGAGTAGTACTG-3’<br>Reverse: 5’-CTGATTCAGTTCTGCACGCTCCTCCTCTTCCTCC-3’
11	pcDNA3-HA-RAGE(S399A)	HA	N/A	Forward: 5’-**GCA**AGTACTGGAGGGCCTTGAGAATTTG-3’<br>Reverse: 5’-CTCGCCTGCCTCAGGTTCCTCCG-3’
12	pcDNA3-HA-RAGE(S400A)	HA	N/A	Forward: 5’-**GCA**ACTGGAGGGCCTTGAGAATTCTG-3’<br>Reverse: 5’-ACTCTCGCCTGCCTCAGGTTCCT-3’
13	pcDNA3-HA-RAGE(T401A)	HA	N/A	Forward: 5’-**GCA**GGAGGGCCTTGAGAATTCTGCAGATATCC-3’<br>Reverse: 5’-ACTACTCTCGCCTGCCTCAGGTTCCTCCG-3’
14	pcDNA3-HA-RAGE(ΔCT)	HA	N/A	Forward: 5’-GAATTCTGCAGATATCCATCACACTGGCGG-3’<br>Reverse: 5’-GATGACCCCAATGAGCAGGGCGGCTGTC-3’
15	pcDNA3-HA-RAGE(ΔD)	HA	N/A	Forward: 5’-GAGGAAGAGGAGGAGCGTGCAGAACTG-3’<br>Reverse: 5’-GATGACCCCAATGAGCAGGGCGGCTGTC-3’
16	pcDNA3-HA-RAGE(ΔDCT)	HA	N/A	Forward: 5’-GAATTCTGCAGATATCCATCACACTGGCGG-3’<br>Reverse: 5’-CTGGTTTTCTGGGGCCTTCCTCTCCTCTCC-3’
17	pECFP-N1-RAGE	ECFP	*Xho* I<br>*Kpn* I	Forward: 5’-ATCTCGAGATGGCTGCCGGAACAGCAGTTGGAG-3’<br>Reverse: 5’-ATGGTACCGCAGGCCCTCCAGTACTACTCTCGC-3’
18	pEYFP-N1-SLP76	EYFP	*Xho* I<br>*Bam*H I	Forward: 5’-TACTCGAGATGGCACTGAGGAATGTGCCCT-3’<br>Reverse: 5’-TAGGATCCGCTGGGTACCCTGCAGCATG-3’
19	pEYFP-N1-SLP76(ΔSAM)	EYFP	N/A	Forward: 5’-AGGAGCATCTTCACACGCAAACCCCAAGTCC-3’<br>Reverse: 5’-CTCTGAGCGAAAGGGCACATTCCTCAGTGCCAT-3’

Supplementary Table 3. Sequences of primers for qRT-PCR.

Gene name	Forward primer	Reverse primer
Tnf	5’-CATCTTCTCAAAATTCGAGTGACAA-3′	5′-TGGGAGTAGACAAGGTACAACCC-3′
Il6	5′-TTGCCTTCTTGGGACTGATGCT-3′	5′-GTATCTCTCTGAAGGACTCTGG-3’
Il1b	5’-GCACTACAGGCTCCGAGATGAA-3’	5’- GTCGTTGCTTGGTTCTCCTTGT-3′
Ccl2	5’-CACTCACCTGCTGCTACTCATT-3’	5’-TGTCTGGACCCATTCCTTCTTG-3’
Ccl3	5’-GCATCACTTGCTGCTGACAC-3’	5’-TTTCTGGACCCACTCCTCAC-3′
Cxcl10	5′-GCCGTCATTTTCTGCCTCAT-3′	5′-GCTTCCCTATGGCCCTCATT-3′
Actb	5’-GAGACCTTCAACACCCCAGC-3’	5’-ATGTCACGCACGATTTCCCT-3′
